# A survey on COVID-19 impact in the healthcare domain: worldwide market implementation, applications, security and privacy issues, challenges and future prospects

**DOI:** 10.1007/s40747-022-00767-w

**Published:** 2022-05-31

**Authors:** Tanzeela Shakeel, Shaista Habib, Wadii Boulila, Anis Koubaa, Abdul Rehman Javed, Muhammad Rizwan, Thippa Reddy Gadekallu, Mahmood Sufiyan

**Affiliations:** 1 School of System and Technology, University of Management and Technology, Lahore, Pakistan; 2 Robotics and Internet of Things Lab, Prince Sultan University, Riyadh, 12435 Saudi Arabia; 3 Department of Cyber Security, PAF Complex, E-9, Air University, Islamabad, Pakistan; 4 Department of Computer Science, Kinnaird College for Women, Lahore, Pakistan; 5 School of Information Technology and Engineering, Vellore Institute of Technology, Vellore, India

**Keywords:** Survey, State-of-the-art, Internet of things (IoT), Internet of medical things (IoMT), Security of healthcare, Application of IoMT, Market trends, COVID-19

## Abstract

Extensive research has been conducted on healthcare technology and service advancements during the last decade. The Internet of Medical Things (IoMT) has demonstrated the ability to connect various medical apparatus, sensors, and healthcare specialists to ensure the best medical treatment in a distant location. Patient safety has improved, healthcare prices have decreased dramatically, healthcare services have become more approachable, and the operational efficiency of the healthcare industry has increased. This research paper offers a recent review of current and future healthcare applications, security, market trends, and IoMT-based technology implementation. This research paper analyses the advancement of IoMT implementation in addressing various healthcare concerns from the perspectives of enabling technologies, healthcare applications, and services. The potential obstacles and issues of the IoMT system are also discussed. Finally, the survey includes a comprehensive overview of different disciplines of IoMT to empower future researchers who are eager to work on and make advances in the field to obtain a better understanding of the domain.

## Introduction

The Internet of Things (IoT) has made remarkable progress supporting many important applications and enhancing the quality of life [1]. IoT devices can collect and understand the surrounding environment. IoT is a very emerging paradigm in the history of the computing field, with an expected 60 billion gadgets at the end of 2022. On the one hand, IoT improves technologies with a very positive impact and enhances different real-time smart applications that improve the quality of the human lifestyle [2]. IoT plays a critical role in improving life by enabling smart things (smart applications), such as smart agriculture, housing, education, transportation, smart manufacturing, irrigation systems, and smart healthcare.

IoT has given people more independence and improved their ability to communicate with others [3]. Novel algorithms and protocols have enabled the IoT to play a key role in global connectivity. It connects many devices to the Internet, including wireless sensors, household appliances, and electrical equipment [4]. Agriculture [5], transportation [6–8], urban [9], the household [10, 11], and healthcare [12–16] are all examples of IoT applications. The Internet of Things is gaining popularity due to higher reliability, reduced costs, and the ability to predict occurrences better. Furthermore, the rapid IoT revolution has been supported by increased awareness of software applications, along with developments in computer and mobile technologies, the ubiquitous availability of wireless technology, and the growth of the digital economy [17]. Actuators, sensors, and other IoT equipment have been connected with other physical devices to control and transfer data using network technologies, such as Zigbee, Bluetooth, WI-FI (IEEE 802.11), and others.

The healthcare industry, in particular, has had rapid expansion in recent years, contributing significantly to employment and revenue [13]. A couple of years back, diseases and anomalies in the human body could only be identified by a medical examination in a hospital. The vast majority of patients were required to remain in the hospital during their treatment. This led to higher medical costs and a strain on rural and distant healthcare services. Due to technological advancements, many diseases may now be diagnosed and monitored using lightweight devices, such as smartwatches.

Furthermore, technological advancements have shifted the healthcare system from a hospital-oriented to a patient-oriented paradigm [18, 19]. Numerous clinical studies could be carried out at home without the support of a physician, such as blood glucose levels, monitoring blood pressure, and pO2 levels. Advanced telecommunications technology can also transmit clinical data from remote places to healthcare centers. The combination of these communication services and rapidly expanding technology (e.g., analysis of big data, machine learning, Internet of things (IoT), cloud and mobile computing, and wireless sensing ) has the potential to transform the industry [20]. The Internet of Medical Things (IoMT) improves electronic medical equipment’s accuracy, dependability, and productivity in the health sector [21–24]. Researchers focus on developing a digital healthcare system by integrating current healthcare resources and services. As the IoT converges in many areas, we are focusing on the research contribution of IoT in the healthcare sector. This article explores how people have participated in the IoMT domain and its applications and future trends in medical services.

Pressure, temperature, electroencephalograph (EEG), electrocardiograph (ECG), and other physiological data are obtained from patients’ bodies using sensors that are implanted or wearable on the human body in healthcare [25, 26]. Humidity, temperature, date, and time are all examples of environmental data gathered. These data assist in developing accurate and appropriate conclusions regarding the health status of the patients. Data accessibility and storage are critical in the IoT ecosystem, because a massive volume of data is captured and collected from multiple resources (sensors, software, applications, e-mail, mobile phones, and others). The data collected by the aforementioned sensing devices are made accessible to doctors, caretakers, and other authorized people. The ability to exchange this data with IoMT practitioners through the cloud/server allows for quick patient diagnosis and, if needed, medical intervention. The majority of IoT systems include a user interface for medical professionals to use as a dashboard, allowing them to control, analyze, and evaluate data. Users, patients, and the communication module must collaborate for successful and secure transmission. The literature [27] has extensively investigated the evolution of the IoT system in medical monitoring, privacy, security, and control. These achievements demonstrate the significance of the Internet of Things in the healthcare sector and its promising future. However, ensuring the quality of service criteria, including sharing information privacy, affordability, dependability, security, and accessibility, is a potential concern while developing an IoMT device.

**Fig. 1:**
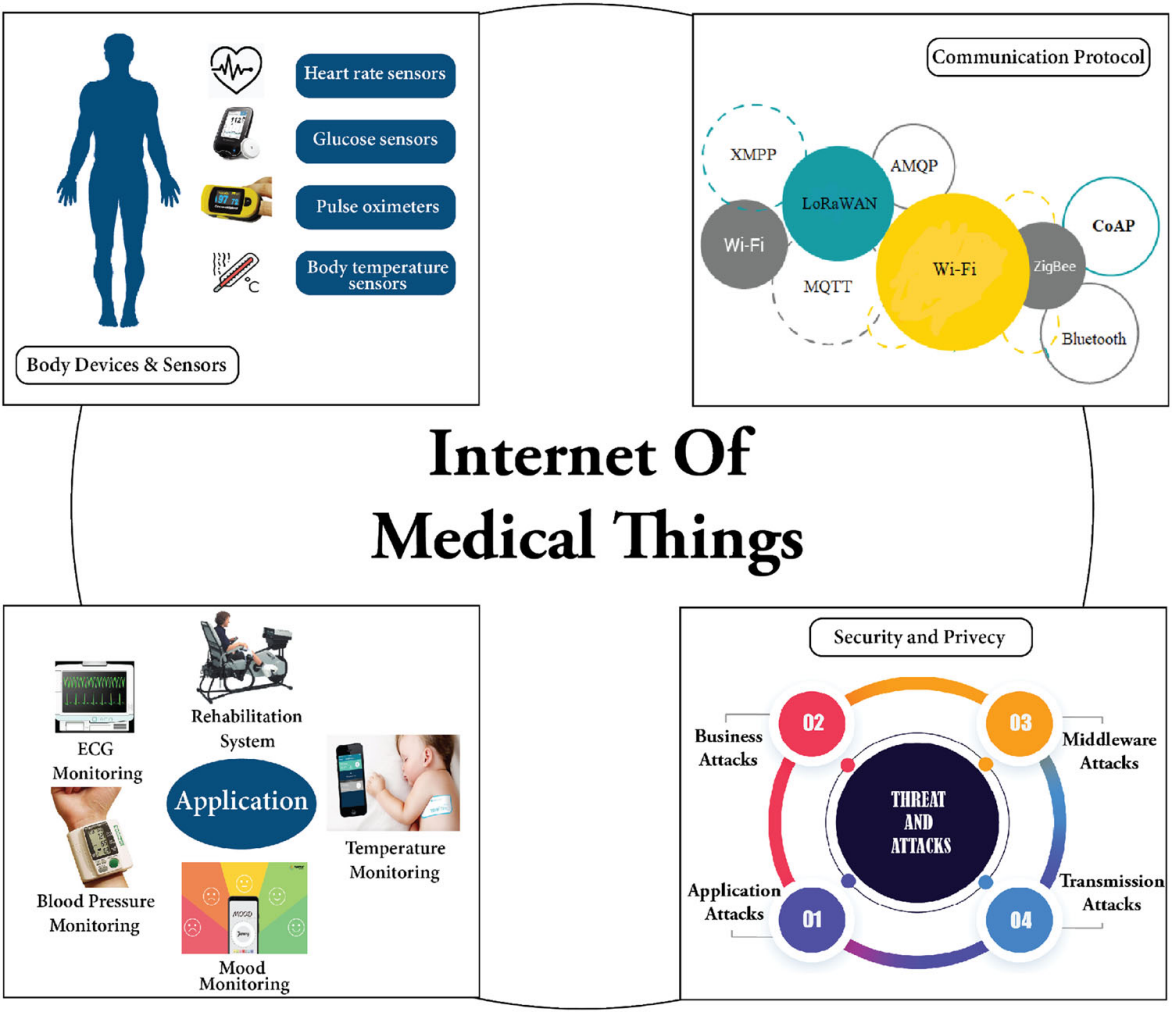
Taxonomy internet of medical things

A wide range of IoT-based applications is developed using IoMT services and technologies. The taxonomy of the IoMT field is depicted in Fig. 1. The physical layer, also defined as the perception layer, involves gathering and transmitting body temperature, heart rate, and other bodily data using physical devices and sensors and then delivering that data to the network layer. It comprises a set of syntactic and semantic rules that determine the operational activity block of a computer network while transmitting data. Numerous protocols [28] have been deployed to ensure efficient data transfer for the IoT infrastructure. According to a recent study, [29], over 90% of all IoT device traffic is unencrypted, exposing 57% of these devices to data breaches.

**Table 1:** Comparison between related surveys

Year	References	Architecture	COVID	Devices	Connectivity technologies	Worldwide market	Security and threats	Applications	Systematic analysis
2019	[30]	IOMT Arc.	X	X	X	X	$\checkmark$	X	X
2020	[29]	IOMT Arc.	X	PD	PD	X	$\checkmark$	PD	X
2020	[31]	X	X	$\checkmark$	$\checkmark$	X	$\checkmark$	X	$\checkmark$
2021	[32]	IOMT Arc.	X	X	X	X	$\checkmark$	X	PD
2021	[33]	IOMT Arc.	$\checkmark$	X	X	PD	$\checkmark$	$\checkmark$	X
2022	[34]	X	X	X	X	X	$\checkmark$	X	X
2022	[35]	PD	X	$\checkmark$	PD	X	X	$\checkmark$	X
2022	This study	IOMT Arc.	$\checkmark$	$\checkmark$	$\checkmark$	$\checkmark$	$\checkmark$	$\checkmark$	Comprehensive systematic review

Key: IoMT Architecture-IOMT Arc., Partially Discussed-PD

The COVID-19 virus has exploded unexpectedly, putting the entire healthcare system on high alert. The Internet of Medical Things (IoMT) and COVID-19 have encouraged scientists to establish a new “smart” healthcare system based on early identification, control of spread, education, and medication, making life in the new norm simpler. The motivation of this survey is to determine the role of IoMT applications in improving healthcare systems as well as technology advancements in IoMT devices, as well as to evaluate the current state of research on the efficacy of IoMT economic advantages to patients and healthcare systems worldwide, as well as to provide a brief overview of technologies that strengthen IoMT and the security and threat challenges that come with developing a smart healthcare system.

In recent years, several significant works have been produced. Researchers from multiple angles have already evaluated this extensive literature to determine the current status of smart healthcare evolution. Thus, a systematic and comprehensive review of related papers published on the subject was conducted. As a result, it is difficult to compare our work to prior surveys. Table 1 presents a fair and comprehensive comparison of prior studies with the proposed study.

This study aims to provide an in-depth assessment and analysis of technology innovations in the smart healthcare system. As the concept of “smart healthcare” evolves with the advancement of associated technologies, there is a need to compile the work from these diverse aspects and provide a comprehensive repository. The significant contributions of this paper are summarized as follows:

- To our knowledge, this is the first survey that provides a comprehensive comparison of the IoT and healthcare domains based on previously published research.
- The paper presents an outline of the future smart healthcare system, its tremendous ability to transform people’s lifestyles, and smart solutions to situations, such as COVID-19. Analyzing the current state-of-the-art development of IoMT worldwide also emphasizes the demands to accomplish the suggested theory.
- The paper discusses the commercially available devices, applications and services, and communication protocols for the Internet of Medical Things.
- The paper highlights IoMT security and privacy challenges and future research directions for smart healthcare systems.

The following is the structure of this paper: “Research methodology” covers the research methodology. “Worldwide IoMT implementation and supportive market” provides an overview of global IoMT implementation and a supportive market. “Applications on internet of medical things” discusses IoMT applications. “IoMT architecture system” addresses IoMT devices and technologies. “IoMT security and privacy” addresses IoMT security and privacy. “IoMT research open concerns and future directions” provides the open issues and future directions. Finally, “Conclusions” concludes the paper. Table 2 shows the lists of acronyms.

**Table 2:** List of acronyms

Notations	Meanings
6LoWPAN	IPv6 over low-power wireless personal area networks
AAL	Ambient assisted living
AMPQ	Advanced message queuing protocol
BLE	Bluetooth low energy
BP	Blood pressure
CAGR	Compound annual growth rate
CoAP	Constrained application protocol
COVID-19	Coronavirus Disease of 2019
CNN	Convolution neural network
CT	Computed tomography
CSMA-(CA)	Carrier sense multiple access with collision avoidance
CSS	Chirp spread spectrum
CSRF	Cross-site request forgery
CRT	Cardiac re-synchronization therapy
DoS	Denial-of-service
DSSS	Direct sequence spread spectrum
EMG	Electromyography
ECG	Electrocardiography
ECIEDs	Embedded cardiac implantable electrical devices
FDA	Federal Drug Administration
GPRS	General packet radio service
GTS	Guaranteed time slot
HTTP	Hypertext transfer protocol
ICD	Implantable cardioverter defibrillator
IDS	Intrusion detection systems
INEs	Implantable nerve electrodes
IoT	Internet of things
IoMT	Internet of medical things
IPs	Internet protocols
LED	Light-emitting diode
LVAD	Left ventricular assist device
LoRaWAN	Low-power wide-area network
Lora	Long-range
LVAD	Left ventricular assist device
M2M	Machine-to-machine
MAC	Medium access control
MIMO	Multiple input multiple output
MQTT	Message queue telemetry transport
MRI	Magnetic resonance imaging
NFC	Near-field communication
NFV	Network functions virtualization
PAN	Personal area network
PHI	Protected Health Information
PHY	Physical
PRISMA	Preferred reporting items for systematic reviews and meta-analyses
PPG	Photoplethysmography
SDN	Software defined networking
RFID	Radio frequency identification
SQL	Structured query language
TCP	Transmission control protocol
UDP	User datagram protocol
UHF	Ultra-high frequency
UWB	Ultra-Wideband
WSNs	Wireless sensor networks
XMPP	Extensible messaging presence protocol
XSS	Cross-site scripting

## Research methodology

**Fig. 2:**
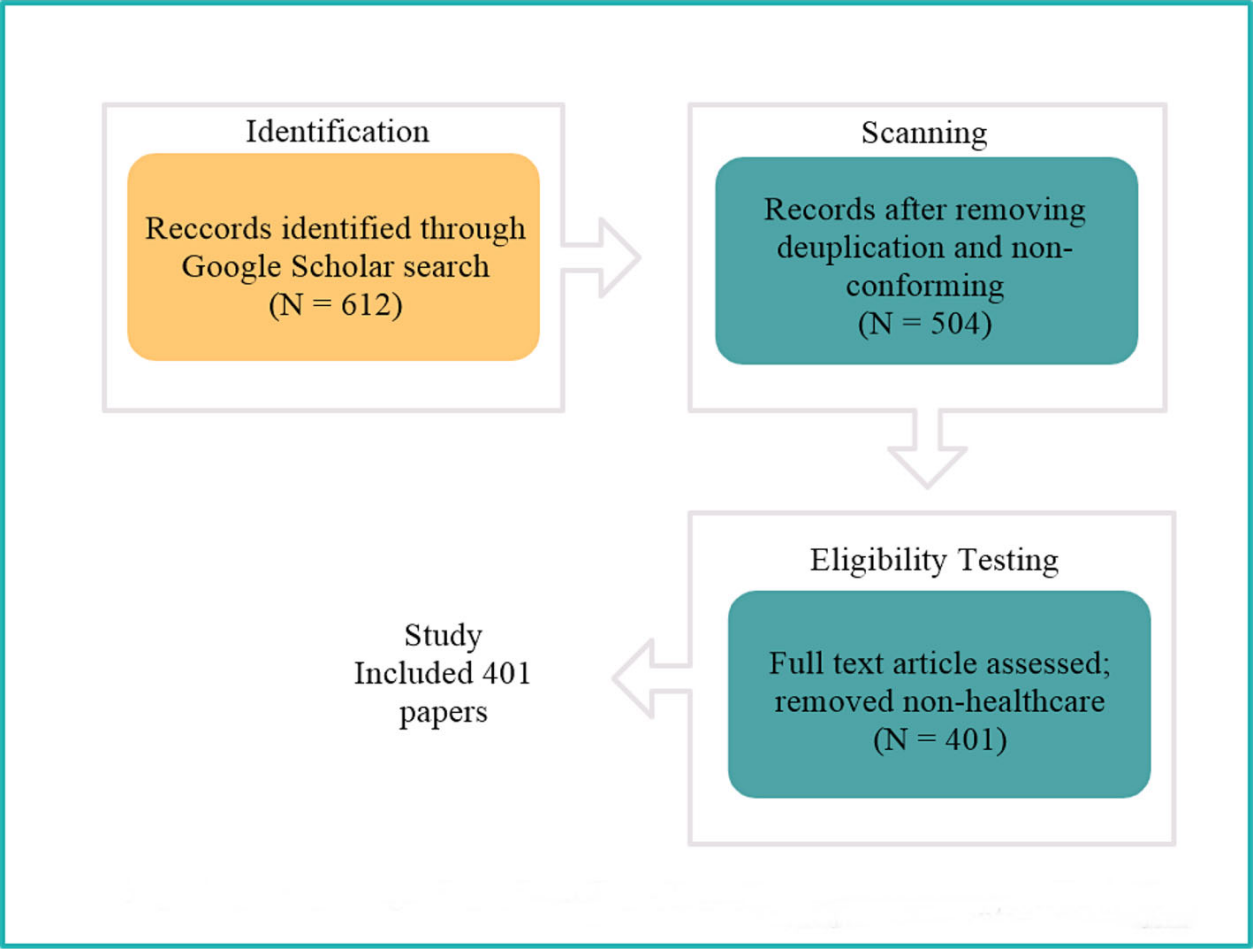
PRISMA systematic review process

The Preferred Reporting Items for Systematic Reviews and Meta-Analyses (PRISMA) systematic literature methodology is selected to find publications and target specific data for this research, as illustrated in Fig. 2, [36]. In October 2019, as part of a research strategy, a search of the Scopus database was conducted using the website’s search tool. Furthermore, a new search in the same database will be performed in June 2020 to include papers published in 2020. The database’s selection is based on its effectiveness. This database is valuable and important in academics, because it contains a variety of publications and archives. IEEE, ACM, and Elsevier are some of the most well-known publishers, aside from being frequently referred to in similar bibliographic reviews [37].In addition, a new search in the same database was constructed in February 2020. The technique for finding study subjects in this database is to look for phrases related to the usage of technology in the field of healthcare, such as “Internet of Medical Things,” “medical devices,” “communication technology,” “smart health care” and other terms associated with “IoT” and “synonym terms. The papers’ publication dates were not criteria for eliminating them. The study is restricted to open-access publications, such as journal and conference articles. This survey article involves creating a search methodology and data source selection and using a collection of keywords. The following keyword combinations have been used as search instructions for the database:

*(“Internet of Medical Things” OR “Applications Using the Internet of Medical Things “OR “Supportable Market Devices” OR “Internet of Medical Things Devices & Protocols” OR and “Internet of Medical Things Security and Privacy.”) AND (LIMIT-TO(ACCESSTYPE(OA)))*

The three parts of the PRISMA review process are identification, scanning, and eligibility testing. In the identification stage, documents are found using a Google Scholar search; 612 papers were found due to this step. Duplicate and non-conforming papers were removed during the scanning process, and 504 were chosen. The papers unrelated to healthcare were then filtered out during the eligibility testing stage. In the last step, 401 papers were selected for this research.

**Fig. 3:**
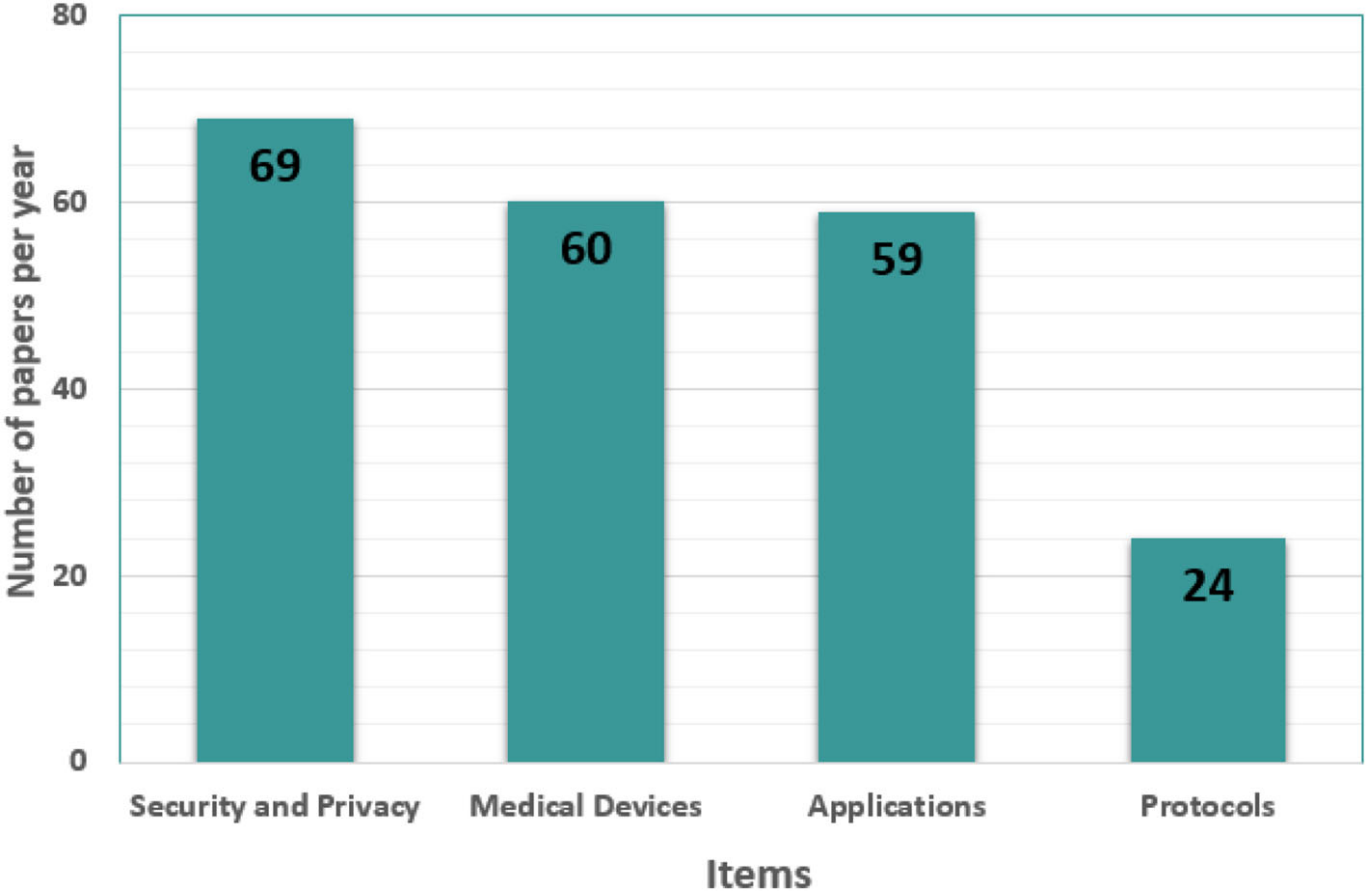
Number of publications published by item

**Fig. 4:**
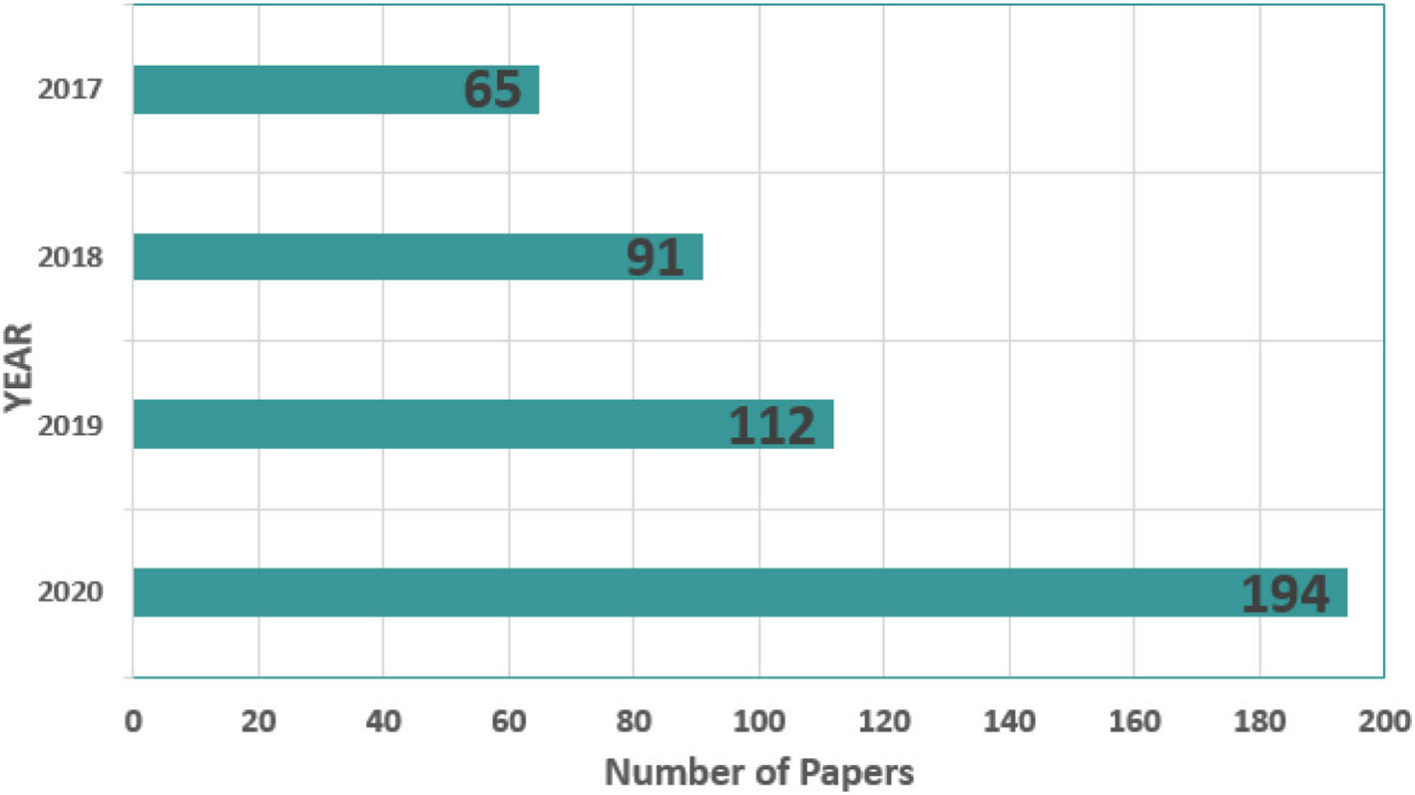
Number of publications per year published by items

Following the search, Fig. 3 depicts the number of publications produced by each search technique (item). The search technique is developed depending on the content of the primary study areas. Figure 4 illustrates a restricted search of publications published between 2017 and 2020. For relevant papers, IEEE Xplore, Google Scholar, ReseachGate, ScienceDirect, the ACM Digital Library, MDPI, and other engineering and health journals were searched. In the first search, 612 papers were found. The phrase “Internet of Medical Things” received the most publications. After eliminating identical and irrelevant entries, the search is narrowed to 401 papers.

## Worldwide IoMT implementation and supportive market

**Fig. 5:**
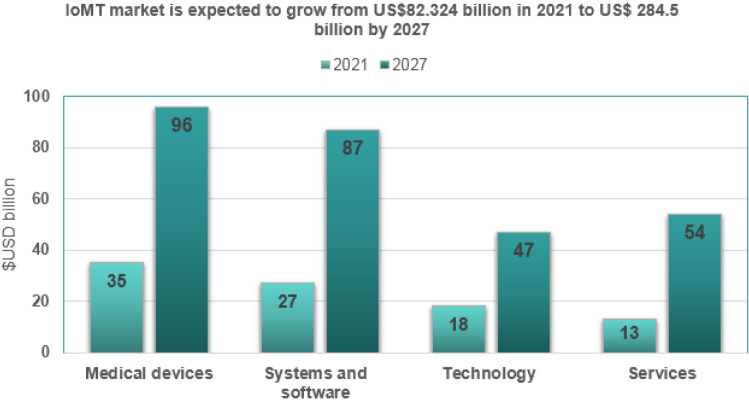
Forecasting the growth of the IoMT market

UnivDatos Market Insights recently added a comprehensive analysis of the internet medical products market to its vast database [38]. The market research on the IoMT is prepared by compiling information on industry factors, such as market drivers, constraints, and opportunities. This novel study combines several investigations to acquire broader market knowledge of internet-connected medical devices. The market research on the IoMT provides a comprehensive analysis of recent industry breakthroughs and market trends propelling market expansion. In addition, this statistical market research resource investigates and predicts the global and regional markets for internet-connected medical devices. Between 2021 and 2027, the international market for the IoMT is expected to develop at a compound annual growth rate (CAGR) of 18.5%, reaching US $284.5 billion, as shown in Fig. 5.

The IoMT uses communication networks to connect medical equipment and applications to a health information technology infrastructure. The IoMT comprises smart wearable, portable smart screens, and other smart health status monitoring equipment. According to a survey [13], over 27% of healthcare enterprises plan to integrate IoT by 2021, while over 60% have already done so. Some of the advantages of IoMT encompass better disease management, better patient outcomes, better therapy and prevention, and better management of health care data and drugs. Furthermore, due to the growth in integrated medical equipment and technological advancements, the industry is experiencing an increase. According to research, by 2021, there will be over 50,000 healthcare innovations accessible. Furthermore, it is predicted that the IoMT would save the healthcare industry $300 billion per year by improving drug adherence and remote patient monitoring.

### COVID-19 impact on IoMT

As a result of the outbreak of COVID-19, the healthcare industry is under tremendous strain and suffering significant change [1, 39–42]. Because of a limitation in capacity in healthcare institutions and end-users need for counseling and treatment via digital media to avoid infection and viral transmission, the demand for IoMT has increased. Furthermore, the government’s focus on remote patient monitoring and the demand for healthcare services stimulate the sector’s expansion. In addition, the healthcare services provider has announced an increase in funding for associated medical research in light of the COVID-19 pandemic [43, 44]. According to the survey, linked medical devices would account for around 48% of all medical devices in 2021, growing to approximately 68% by 2027. Furthermore, the organizations that manufacture linked devices have declared an increase in their R & D (research and development) budgets. R & D spending on connected medical devices would have increased to around 42% by 2025, up from around 34% in 2020.

### IoMT market segments

**Fig. 6:**
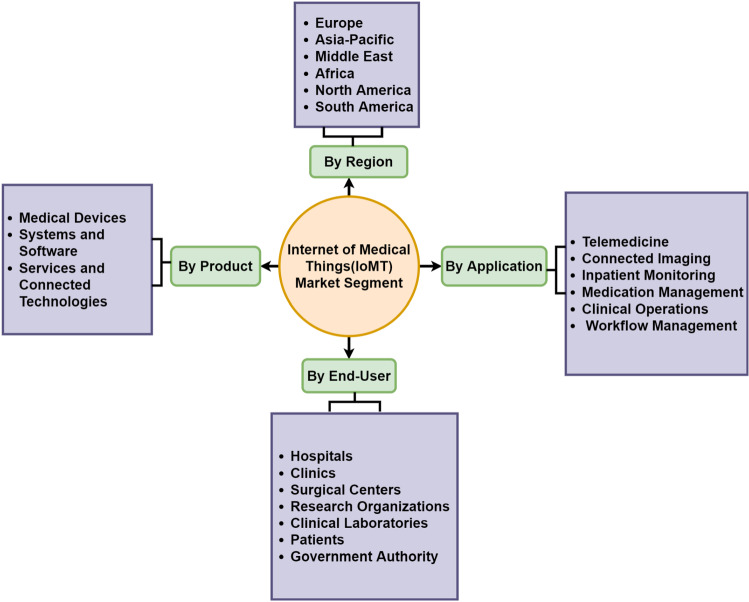
Taxonomy of IoMT by market segments

The expanding digitization of healthcare systems is pushing this trend to enable higher quality care, the increased need for digital healthcare devices, and rising demands from an elderly society and those suffering from severe diseases. The IoMT market study is thoroughly researched and includes various factors that will assist stakeholders in making better-informed decisions [38]. It is projected to grow at a 30.8% CAGR(Compound annual growth rate) from $82 billion in 2021 to $284.5 billion in 2027 [45]. As shown in Fig. 6, the IoMT market segment is separated into four categories that may be viewed from the perspective of a component, region, end-user, or application.

#### IoMT region

**Fig. 7:**
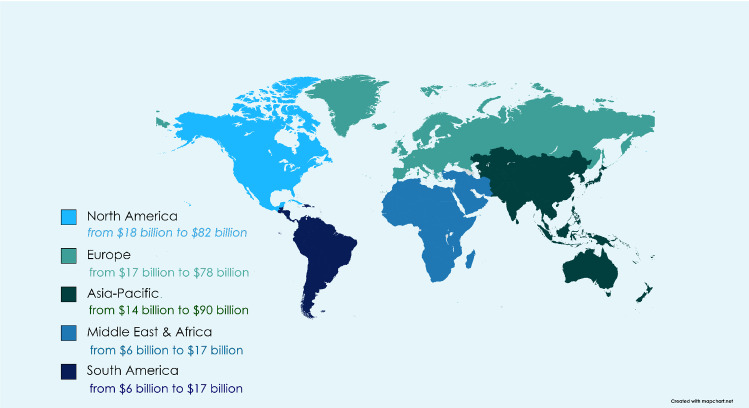
CAGR from 2021 to 2027 on a worldwide scale

The geographical segments of the IoMT market include Europe, Asia–Pacific, the Middle East, Africa, North America, and South America. Due to developments in the healthcare industry and increased healthcare information and technology (IT) investment across nations, North America accounted for $18.3 billion, or 33% of the overall IoMT market in 2020, followed by Europe with $17.4 billion, Asia–Pacific with $14.0 billion, the Middle East and Africa with $6.4 billion, and South America with $6.1 billion, as illustrated in Fig. 7. Because of the greater extent of unmet demand and the increasing demand for general hospitals and medical centers being developed in this region, the Asia–Pacific IoMT market is expected to grow at the fastest rate, with a CAGR of 34.3% during the forecast time frame [45].

#### IoMT product

**Fig. 8:**
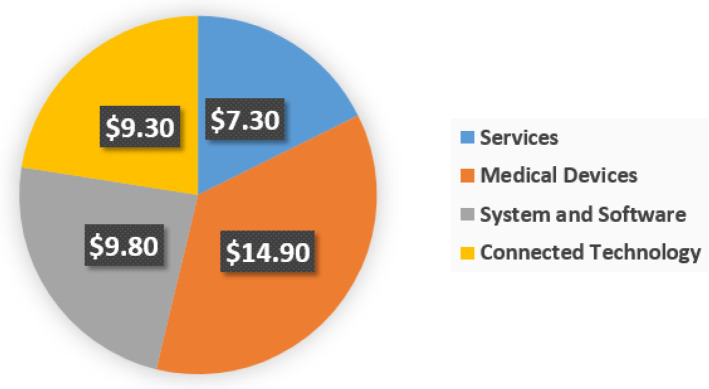
Market segmentation by product [46]

The market is divided into four product component categories: services, medical devices, systems and software, and connected technologies, as depicted in Fig. 8. The medical devices sector has the largest market share among product categories, and it is expected to grow at a CAGR of 16.9% to reach US $93.6 billion by 2027.

- *Medical devices* Medical Devices are classified into three types. Wearable External Medical Devices, Implanted Medical Devices, and Stationary Medical Devices. Wearable external medical devices account for most medical devices and are expected to grow at a CAGR of 18.7% between 2021 and 2027. Wearable external medical devices accounted for approximately 27.5% of total sales in 2019 [38].
- *Systems and software* Data analytics, network security, application security, remote device management, and network bandwidth management. The market is segmented into five divisions based on systems and software. The remote device management category captured the largest market share IoMT market in 2019, accounting for around 52.3% of the market share, and it is anticipated to rise by 42.5 billion USD by 2027.
- *Services* The market comprises three service segments: consulting, support and maintenance, and deployment and integration. Support and maintenance have the largest proportion of all service categories, and they are predicted to grow at an 18% CAGR from 2021 to 2027. In 2019, the revenue share of the support and maintenance sector was 47% [46]
- *Connected technologies* The IoMT ecosystem’s frameworks are connection technologies that connect people and things to the Internet. Wireless technologies used in health care include WiFi, BLE, Near-field communication (NFC), Zigbee, cellular, and satellite systems. Continuous wireless communications are made possible by compatibility between wireless technologies, lower power consumption, and spectrum extension. In 2017, the connectivity technology market was worth $9.3 billion, and it is predicted to grow to $28 billion in 2022 and $34 billion in 2027 [46].

**Fig. 9:**
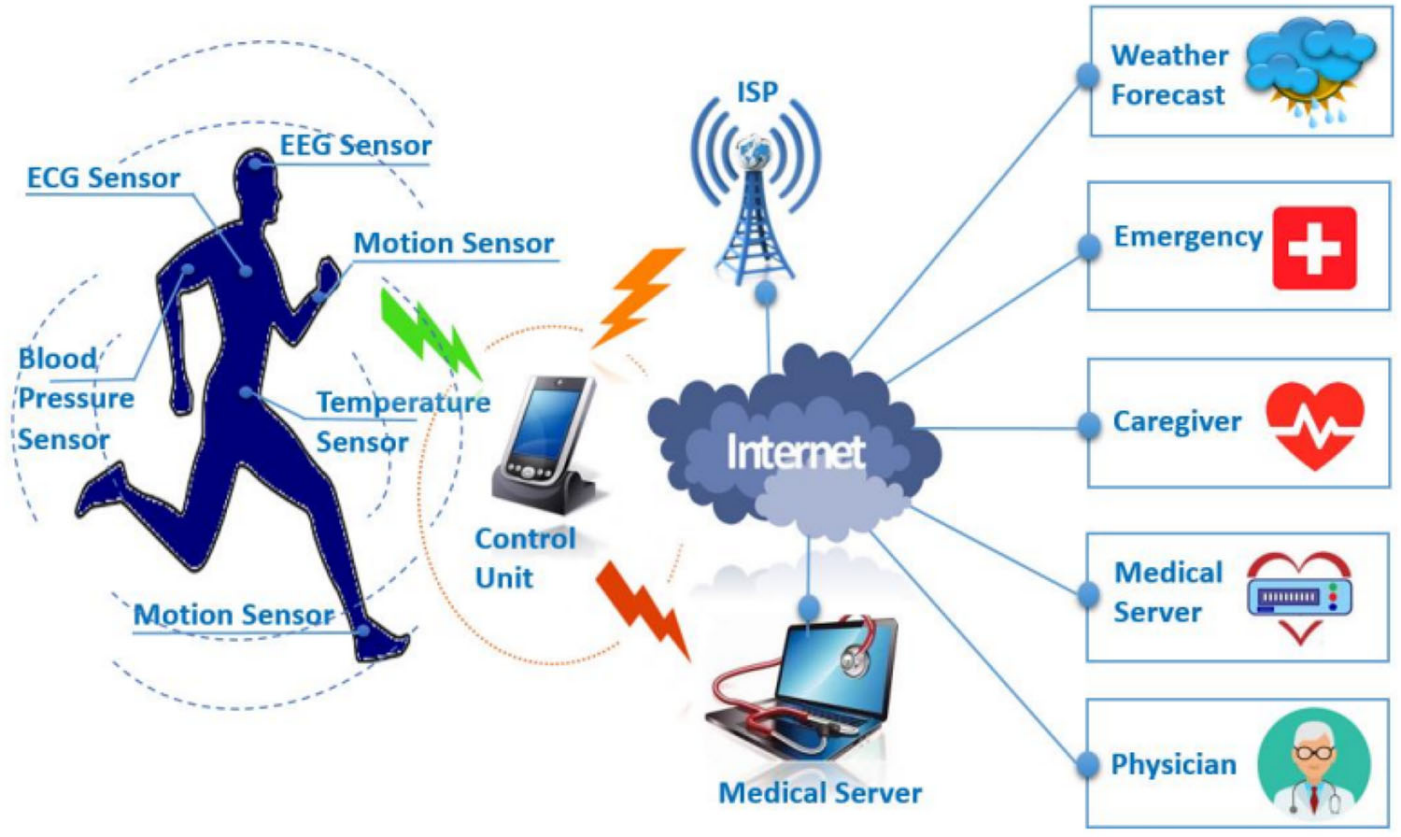
End-user of IoMT applications

#### IoMT applications

Applications have segmented the IoMT market. The application services available include telemedicine, connected imaging, inpatient monitoring, medication management, clinical operations, workflow management [47–49]. Telemedicine dominated the global IoMT market in 2019, accounting for approximately 31.2% of the market, and revenue is expected to reach $78.6 billion by 2027 [38]. In “Introduction”, the IoMT applications are thoroughly discussed.

#### IoMT end-user

Hospitals, clinics, surgery centers, clinical research organizations, and other medical organizations are among the largest end-users in the IoMT market,as shown in Fig. 9, with a 17.3% CAGR from 2021 to 2027. In 2019, hospitals, surgical centers, and clinics generated 74.9% of total revenue. [38].

## Applications on internet of medical things

**Fig. 10:**
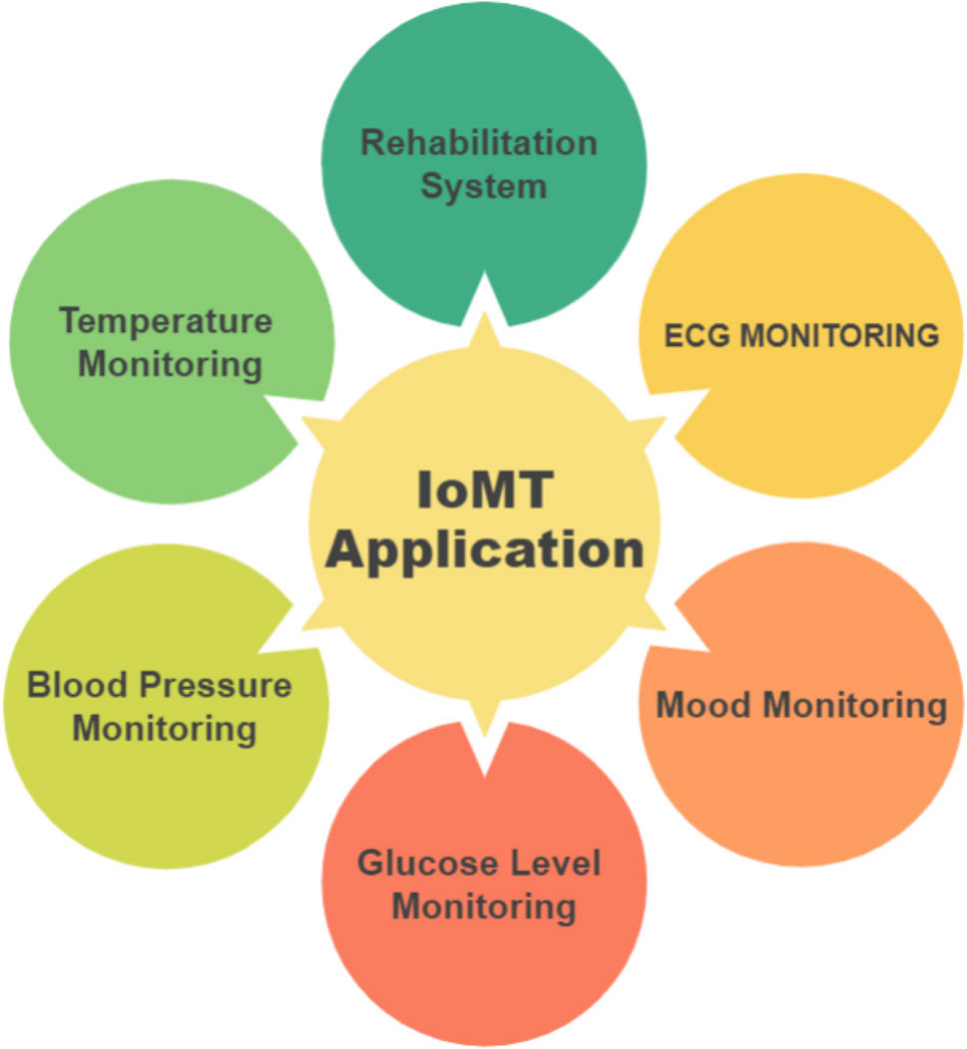
Category of IoMT applications

A wide range of IoT-based applications is built using IoMT services and technologies [50]. Many suggestions have been made for the progress of humanity by researchers in various fields. To put it another way, concepts are more focused on the developer, whereas applications are more focused on the user. Fast improvements in IoT technology have made portable gadgets, wearable sensors, and medical equipment more affordable and user-friendly. These technologies might gather clinical information, make disease predictions, track patients’ health, and send out notifications in a medical emergency. Some of the most recently available commercial devices and applications are discussed in the following section. Various IoMT-based applications, including single and multiple condition applications, as shown in Fig. 10, have also been addressed.

### Rehabilitation system

IoT is used in treatment in various forms, such as curing strokes, cancer, injuries, and other physical impairments. Physical therapy can help a patient regain functional capacity in conjunction with rehabilitation. The rehabilitation process includes diagnosing the issue and assisting patients in resuming their regular lives. In [51, 52] presented a smart walker rehabilitation system that employed a multi-modal sensor to monitor the patient’s walking pattern and analyze movement parameters. The smart walker tracked several movement parameters, such as force, position, elevation, angle, etc., while a patient used it.

The physicians accessed this data and generated diagnostic reports using a smartphone app. Furthermore, by combining a smart wearable wristband, a robotic hand, and a machine learning algorithm, a stroke recovery system was constructed [53] A low-power IoT-based textile electrode was used to create the armband, which can monitor, reprocess, and send the bio-potential signal. During the post-stroke healing period, the 3D-printed robotic arm also monitored muscle activity and aided the patient in correcting their motion pattern [53]. Another study [54] described a sports rehabilitation system that tracked posture, temperature, mobility, electromyography (EMG), and electrocardiography (ECG), as well as provided feedback to the sportsman. Healthcare experts could utilize the information acquired to anticipate patients’ recuperation and develop treatment plans.

### ECG monitoring system

An electrocardiogram (ECG) exhibits the re-polarization and depolarization of the ventricles and atria, indicating heart electrical activity. An electrocardiogram (ECG) is a form of an electrocardiogram that presents the essential heart muscle beats and can detect a variety of cardiac disorders.

Arrhythmia, a prolonged QT interval, myocardial ischemia, and other abnormalities. IoMT technology for ECG monitoring for early detection of heart issues has shown potential. In the past, IoMT has been used in ECG monitoring in several studies [54, 55]. A receiving processor system and a wireless data collection system are presented in the study [55], which is an IoMT-based ECG monitoring system. It used a real-time search automation approach to diagnose cardiac abnormalities in real-time. A tiny, low-power, wearable ECG monitoring device combined with a t-shirt collected high-quality ECG data using a bio-potential chip. The collected information was then transmitted to the end-users via Bluetooth. A smartphone app might be used to visualize the captured ECG data.

Real-time monitoring in an IoMT system may become feasible when an IoMT system is integrated with big data analytics to handle additional data storage [56]. By combining the concepts of nano-electronics, big data, and IoMT, authors in [57] proposed an ECG monitoring device capable of long-duration monitoring. The suggested technology may run on just 5.2 Millie-Watts of electricity. It is worth noting that the authors of [58] attempted to tackle the problem of power consumption in a wearable ECG monitoring device.

They have suggested compressive sensing as a novel technique for reducing power usage and improving ECG monitoring performance. The study [59] describes an IoMT-based ECG monitoring and fall detection system that employs a mobile application and a cloud-based server. This device was created to provide elderly patients with real-time monitoring by constantly reviewing their ECG and accelerometer data.

### Temperature monitoring system

The temperature of the human body is a significant consideration in many medical diagnostics as it indicates homeostasis. Keeping track of temperature changes over time in various disorders helps clinicians make conclusions about the patient’s health. In some instances, such as trauma and infection, a change in body temperature may indicate. A temperature thermometer placed in the ear, mouth, or abdomen is the most commonly used device for measuring temperature. The patient’s discomfort and the high risk of infection, on the other hand, are always a consideration with these operations.

However, with the subsequent improvement of IoMT-based technology, various solutions to this problem have been proposed. The study [60] presented a 3D-printed wearable gadget that can be put on the ear and uses an infrared sensor to determine inner body temperature from the tympanic membrane. The device contained a wireless sensor device and a data computing unit. The temperature measured in this experiment is unaffected by the surroundings or any other physical activity.

The researcher [61] designed a temperature monitoring system based on IoMT using the Raspberry Pi and Arduino. The temperature readings were saved in a database and viewed on a website accessed through a computer or a smartphone. In another research [54], a lightweight wearable device was used to detect infants’ body temperatures in real-time. It may also notify the caregivers if the temperature exceeds a certain threshold.

### Mood monitoring system

Self-monitoring of one’s emotional state helps one understand one’s mental state. Mood monitoring is used to sustain a healthy state of mind by providing essential information about an individual’s emotional state. It also helps healthcare providers manage mental diseases, such as depression, stress, and bipolar disorder. In [62, 63], the authors proposed a mood mining technique that employed convolution neural networks (CNNs) to assess and categorize an individual’s mood into six categories: cheerful, enthusiastic, sad, peaceful, disturbed, and furious. A similar study used an interactive technology called “Meezaj” to achieve real-time emotion monitoring. The software also highlights the significance of emotions in decision-making and supports policymakers in identifying the key factors that influence a person’s happiness. Because of implementing an advanced machine learning system, stress may now be diagnosed in advance using heart rate. Furthermore, the device may interact with the patient regarding their degree of stress [64]. It is also worth noting that analysis of stress can be useful in developing an IoMT-based system responsible for preventing an incident.

The research proposed in [64] developed a wearable sensor that can assess four unpleasant emotions or moods in a person or driver: sorrow, anger, tension, and fright. Smart technology determines whether or not the driver is in a subconscious state by analyzing the variance in these emotions. When a driver enters a hypnotic state, the technology disables the vehicle’s DC motor.

### Blood pressure monitoring

One of the mandatory processes in any diagnostic procedure is measuring blood pressure (BP). Most blood pressure testing protocols require at least one person to participate. In contrast, IoMT and other sensor technology have improved how blood pressure is traditionally measured. In [65], for example, a wearable cuff-less device that can monitor both diastolic and systolic pressure was presented. The data collected can be saved in the cloud. The device’s effectiveness was also examined on 60 people, and its validity was confirmed. In his IoMT-based BP measuring system, Guntha [65] used fog and cloud computing.

The system was ready to be monitored in real-time for an extended period. The gadget may also save the collected data for future reference. A deep learning model based on CNNs that incorporates time-domain features was used in a similar study [66] to assess systolic and diastolic blood pressure. In [67] proposed using an ECG signal plus a photo-plethysmo-gram (PPG) collected from the fingertip to measure blood pressure. The attached micro-controller module was used to calculate the BP, and the data was then sent to cloud storage.

### Glucose level monitoring system

Diabetes is a chronic disease in which the blood glucose levels in the body are persistently and exceptionally high for a long time. It is among the most common human ailments. The three most frequent types of diabetes are type I, type II, and gestational diabetes. An oral glucose tolerance test, a fasting plasma glucose test, and a random plasma glucose test can all be performed to determine the disease and its kind. However, the most popular technique is “finger-picking,” followed by blood glucose level testing.

Many non-invasive, pleasant, affordable, and efficient wearable devices for blood glucose monitoring have been devised using recent developments in IoMT technology [68, 69]. The research [70] presented a non-invasive m-IoMT-based glucometer for real-time glucose level monitoring. Alarcon Paredes [71] developed a blood glucose measurement glove equipped with a Raspberry Pi camera and a visible laser beam. The wearable devices were linked to the healthcare experts via an IPv6 connection. A sequence of photos taken of the patients’ fingers was used to determine their diabetes status. In a separate study [72], In an IoT ecosystem, a procedure based on a double moving average was employed to monitor glucose levels. It is noteworthy that electro-optic sensors, such as near-infrared photo-diodes and infrared LEDs were employed to track glucose levels. The laser beam reflected from the body is used to calculate the body’s glucose level [73].

### Other notable applications

IoMT provides numerous applications that are not constrained by the original characteristics. The variety of IoT applications is continually increasing due to rapid technological improvement. Some research domains that did not reveal the fusion of IoT devices are now effectively adopting this technology. Cancer therapy, remote surgery, aberrant cellular development, hemoglobin testing, and other procedures may be included. A novel IoT-based cancer treatment framework was presented in [74], which integrated several stages of cancer treatment, such as radiation and chemotherapy.

A smartphone application was used for online consultations with the physicians. The laboratory reports of patients were saved on the cloud server and retrieved by a medical professional to determine when and how much medicine to provide. Another possible use is the diagnosis of lung cancer with an IoT-based system employing different cutting-edge machine learning algorithms [75, 76]. Furthermore, a recent study [77] showed that an IoMT-based system might be used to identify skin lesions. Cecil et al. [78] used IoMT in the development of a next-generation surgical training system.

The gadget uses virtual reality to provide a teaching environment and a platform for surgeons worldwide to engage. A human–robot collaboration system capable of performing minimally invasive surgery has been suggested in [79]. Hemoglobin levels in the blood may be measured using a portable instrument [79]. The gadget used a light-emitting diode (LED), photo-plethysmography (PPG) sensors, and photo-diodes for hemoglobin measurement. The device’s effectiveness was further confirmed by comparing the findings to a well-known colorimetric test. Table 3 has highlighted several of the IoMT applications.

**Table 3:** List of IoMT applications

References	Application/task	IoMTs
[80]	Smart rehabilitation system	BCI-actuator, brain EEG cap, motion sensor
[54]	Disease detector	Sensors and boards
[81]	Action and activity recognition for health monitoring	Wearable camera, google glass, motion wrist sensor
[82]	Heart rate estimates	PPG, ECG, wearable sensors
[83]	Sleep pose recognition	Depth and RGB sensors, Carmine camera
[84]	CT-scan images similarity measures	SPET-Images, CT-MR, CT-MR t1, CT-MR t2
[85]	Autistic patient monitoring	Voice pathology, cloud technology
[82]	Stress evaluation	PPG, ECG, wearable sensors
[86]	Cuff less blood pressure monitor	ECG, photo-plethysmo-gram signals
[87]	Emotion recognition	Respiration, physiological signals, Galvanic skin response, 4-EMG, body temperature
[88]	Sleep apnea detection	Heart-rate, saturation, abdominal, respiratory belt

## IoMT architecture system

**Fig. 11:**
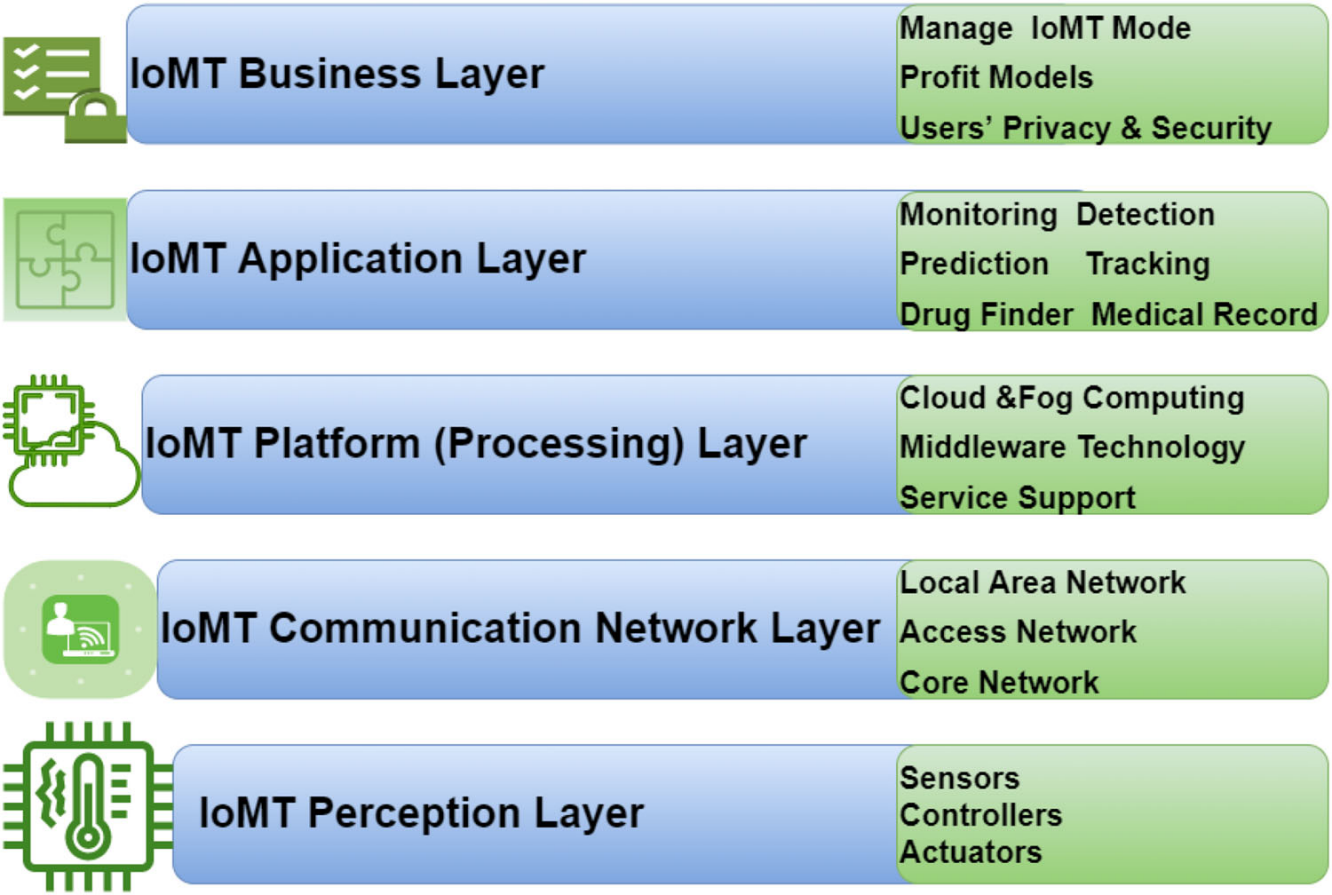
IoMT five-layer architecture model

Depending on the complexity of healthcare systems, it is critical to handle dozens of medical devices connected to the Internet in a heterogeneous manner to maintain the greatest standard of dependability. As a result, an adaptable, layered architecture is required. The IoMT five-layer model describes a tiered architecture, where each layer performs a distinct function [89]. The five layers are perception, communication network, platform (processing), application, and business layers, as illustrated in Fig. 11. The device layer, also known as the perception layer, comprises hardware, including sensors, actuators, and controllers. Connectivity to other smart IoMT devices, network devices, and servers is the responsibility of the communication network layer. Its characteristics are also leveraged in the processing and transmission of sensor data. Sensor data is transmitted from the perception layer to the platform (processing) layer via networks (for example, Bluetooth, RFID, WiFi, 3G, LAN, and NFC). The IoT platform layer, also known as the processing (middleware) layer, stores, analyses, and processes massive volumes of data received from the communication layer and provides service support, interpretations, cloud computing, and middleware technology. It can handle and facilitate many services to the lower layers, such as notifications, data retrieval, big data processing, cloud computing, and analytics for IoMT applications through cloud platform services, such as Microsoft Azure, Amazon Web Services, and Google Cloud, and others. The application layer is in charge of providing the user with application-specific services [90, 91]. This layer contains a variety of devices, including monitoring devices, location devices, tracking systems, telemedicine, medical e-records, health fitness systems, remote diagnostic systems, and so on. The business layer is in charge of the overall IoMT system, including applications, security, business and profit models, and users’ privacy. Each feature has its own set of security and privacy problems. As a result, security and privacy problems are classified into the following categories based on their frequency in each layer.

**Fig. 12:**
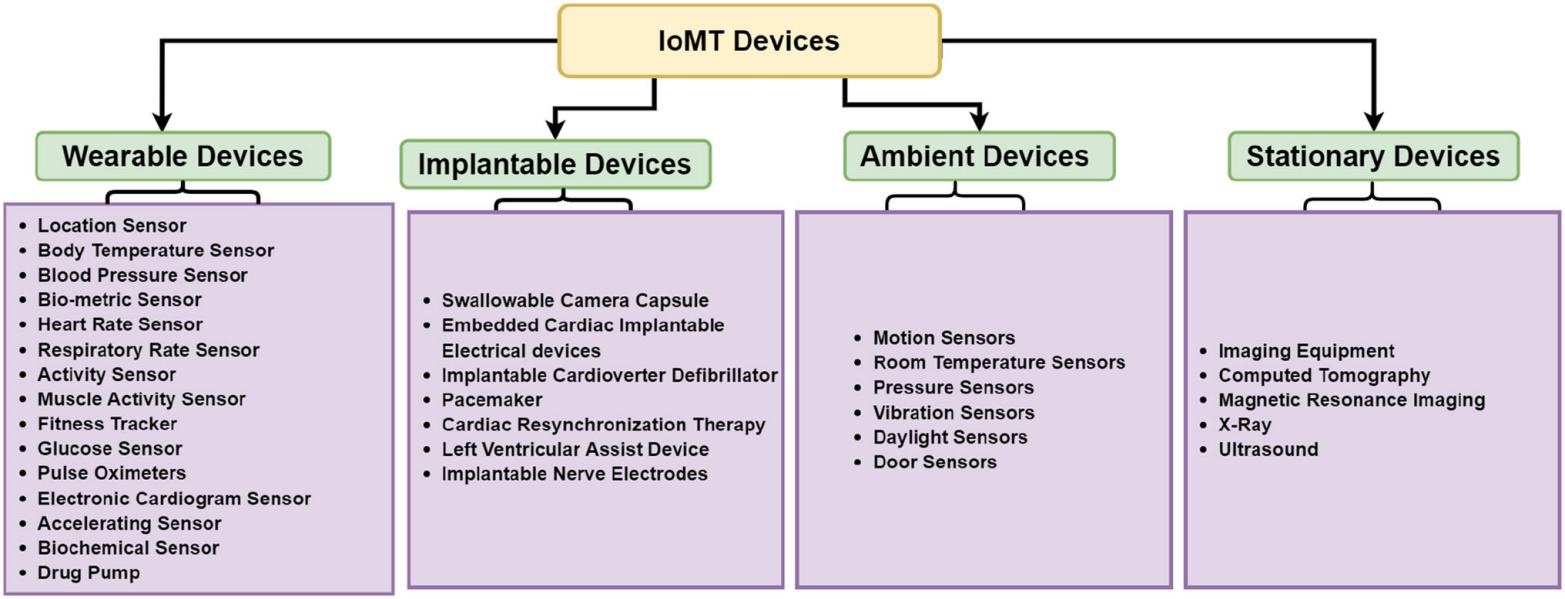
Category of IoMT devices

### Perception layer

The perception layer is in charge of obtaining and gathering data from physical devices, such as sensors, such as heart rate, body temperature, and other physiological parameters, and then sending that data to the network layer. Many sensors, for instance, interact in healthcare monitoring systems to guarantee that the patient has always been monitored and receives assistance as quickly as feasible. This section addresses the interactions of stakeholders, such as doctors and patients, with the four types of medical things, as shown in Fig. 12.

#### Wearable devices

The primary issue of personal health is a concern in this pandemic-influenced period. Many people are concerned about illness prevention, while others look for strategies to stay healthy. The emergence of wearable technology has made it possible to keep track of one’s health. Wearable technologies, such as smartwatches and other devices can provide accurate, continuous, and real-time patient monitoring. Table 4 shows some of the wearable sensors for healthcare that are available on the market.

- **Real time Location sensor (RLTS):** It can be deployed to track healthcare personnel in big hospitals when a patient or other member of staff calls for rescue during a life-threatening emergency.RTLS may track patients’ body actions to guarantee their survival, especially critical for Alzheimer’s and dementia patients. An RTLS can be implemented using various technologies, such as camera vision, light, infrared (IR), ultrasound, sound, GPS, and RFID.
- **Body temperature sensors:** These sensors are used to determine the temperature of the body. The most commonly used temperature sensors are the LM-35, DS18B20,TMP236, and MAX3020 [35]. The LM-35 is most generally used in wearable sensor networks because of its many uses in remote patient monitoring. It has a higher temperature range than the others, ranging from 55 to 150 $^{\circ }$C with an accuracy of 0.5 $^{\circ }$C. A thermistor can be used in place of a temperature sensor, since it is inexpensive, dependable, and waterproof, with a precision of up to 0.25 $^{\circ }$C.
- **Blood pressure sensors:** These devices are used to keep track of a patient’s blood pressure. There has been much research into non-invasive blood pressure monitoring, particularly the oscillometric approach, which uses an air-inflated cuff to pressurize blood vessels and measure systolic and diastolic pressure. In the other approach, biological sensors, such as ECG and PPG sensors are utilized to estimate blood pressure without using a cuff. The traditional method of measuring blood pressure needs many devices and is thus inconvenient. Advances in ECG and PPG signal processing have formed a system for estimating and evaluating patients’ blood pressures.
- **Bio-metric sensors:** Wearable biometric sensor devices may collect and analyze personal data in real-time, such as heart rate and sleep habits. Recent biometric sensors are precise, versatile, and scalable to various devices, such as smartwatches, earbuds, and armbands. Due to technological advancements in microcontrollers (MCUs) and system-on-a-chips (SoCs), continuous glucose monitoring, blood oxygen saturation (SpO$_2$) monitoring, and mood and stress monitoring are all becoming widely widespread and are expected to be widely accepted by the community.
- ** Heart rate sensors:** Electrocardiography (ECG) or photoplethysmography (PPG) measures the heart rate. A PPG is a standard method to monitor heart rate, since it is a simple, non-invasive, and inexpensive optical measurement technology. A PPG sensor comprises a photodetector and a light source placed on the skin’s surface to monitor fluctuations in blood flow, and the second derivative wave of a PPG signal offers vital health information. As a result, evaluating this waveform can assist in diagnosing a variety of cardiovascular disorders. The PPG sensor is most commonly placed on the patient’s earlobe, finger, or forehead.
- **Electronic cardiogram sensors:** Data from an electrocardiogram (ECG) is needed to keep track of a healthy heartbeat and strength. It evaluates the heart’s muscular and electrical performance. It is crucial for predicting and preventing cardiovascular disease. The ECG monitoring device AD8232 is frequently used. It can function as a heartbeat sensor and an ECG graph sensor.

**Table 4:**
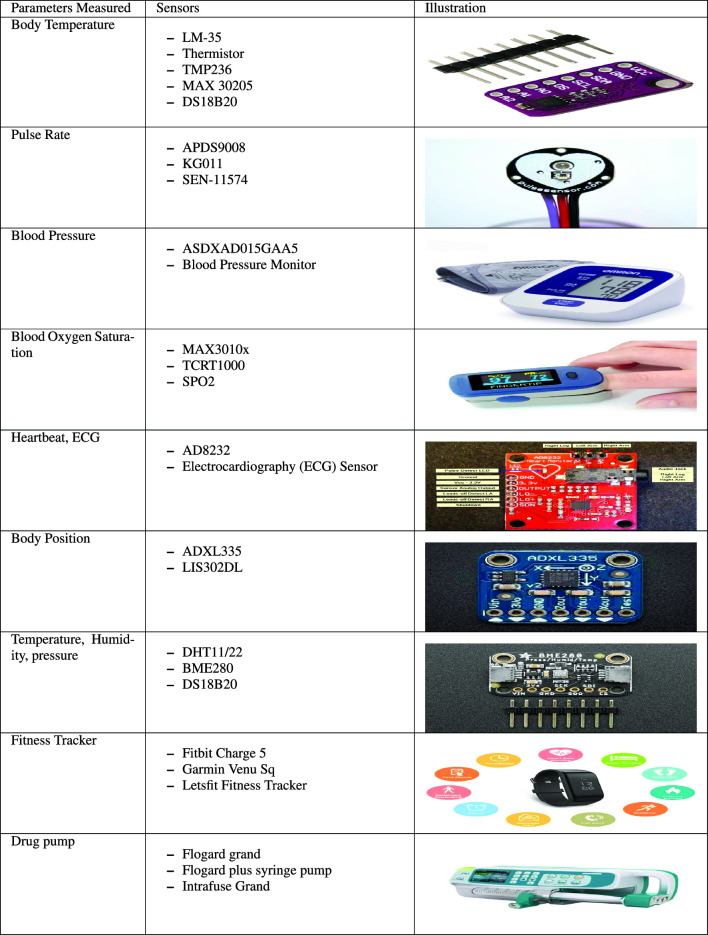
Wearable sensor in the healthcare sector
- **Respiratory rate sensors: ** Measuring respiratory rates is crucial in healthcare systems for identifying a variety of ailments, including pneumonia and asthma. Considering IoT devices must be power efficient, continuously monitoring a patient’s respiration rate is difficult. These sensors keep track of how well patients are breathing. A thermistor is the most common hardware device for monitoring the rate of breathing. A thermistor is less expensive, uses less power, and has a longer lifetime, which is ideal for medical applications.
- **Activity sensors:** Activity trackers record patient movement throughout the day and also at night. For example, Gyroscope sensors track behaviors such as sleeping and jogging. The tracker records the number of steps a patient takes during the day, activity duration, the distance covered, whether they reach their daily target, calorie consumption, and fat burn. At night, the patient’s sleep duration is recorded, and the quality of his or her sleep is evaluated.
- **Muscle activity sensors:** The electromyographic (EMG) sensor is a sensor that detects electrical muscle activity. It is frequently used as a command signal for various prosthetic systems. This sensor allows doctors and medical workers to keep track of a patient’s nerve and muscle issues. These sensors are also deployed in wearable technology to track patients’ behavior. In addition, Advancer Technologies is working on an Arduino-based EMG sensor. The impacted condition of the patient’s health can be determined using this sensor.
- **Fitness trackers:** A wearable fitness tracker uses special gizmos to keep track of various parameters of the individual wearing the tracker. Many different sensors can be added to a fitness tracker, such as a three-axis accelerometer, gyroscope, altimeter, temperature sensor, bioimpedance sensor, optical sensor, etc. These sensors measure several parameters, such as acceleration, frequency, duration, intensity, and patterns in individuals’ movements.
- **Glucose sensors:** Diabetic people need blood glucose monitors to keep their blood sugar levels stable. Blood glucose monitors occur in many forms. On the other hand, non-invasive and smart glucose monitors are preferable in an IoT context to prevent the challenges of invasive blood glucose monitoring and the potential for infection from injections. The Company “DIAMONTECH” has designed an intelligent, non-invasive glucose meter with this in mind. It processes and detects any irregularity in the obtained patient data using near-infrared spectroscopy and machine learning techniques. The information is transferred to the cloud for analysis and reporting. These characteristics enable endocrinologists to take care of their patients from afar.
- **Pulse oximeters:** This is a non-invasive device that measures the oxygen saturation of a person’s blood. This data can help track and diagnose any changes in a patient’s health. Asthma, pneumonia, anemia, lung illnesses, and other health problems can all be detected with a blood oximeter. The MAX30102 is the most widely popular and effective pulse oximeter sensor in smart healthcare. The sensor is a low-power device (1.8 V). Its compact size allows it to be readily fitted into smart wearables or smartphones.
- **Accelerating sensors:** The use of an accelerating sensor to track physical activities helps detect postural orientation and motions in the real world. It is used to keep track of an elderly patient’s recovery.
- **Biochemical sensors:** Wearable biochemical sensors provide much potential for personal medication and continuous health monitoring. This sensor detects and quantifies numerous chemical elements in the human body, such as perspiration, saliva, and tears, to track alcohol and drug consumption. It is also used to identify harmful chemicals in the atmosphere and monitor biochemistry.
- **Drug pumps:** A drug pump is a medical device that controls the delivery of fluids into a patient’s body. It provides predetermined doses of medicine to the patient. Drug pumps can supply large or moderate doses of fluids, and they can be used to deliver nutrition or pharmaceuticals, such as insulin or other hormones, antibiotics, chemotherapeutic drugs, and pain relievers. A “smart drug pump” has safety measures, including user alerts that trigger when a potentially harmful drug interaction appears or when the user adjusts the pump’s settings outside of predefined normal ranges.

**Fig. 13:**
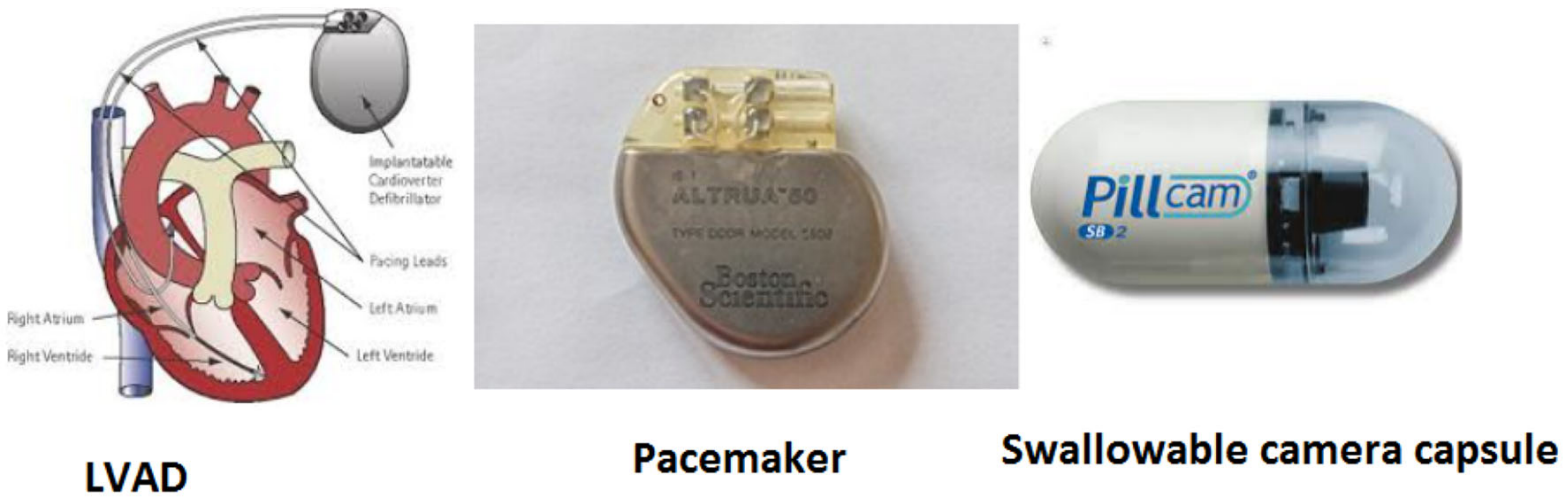
Implantable devices in IoMT domain

**Fig. 14:**
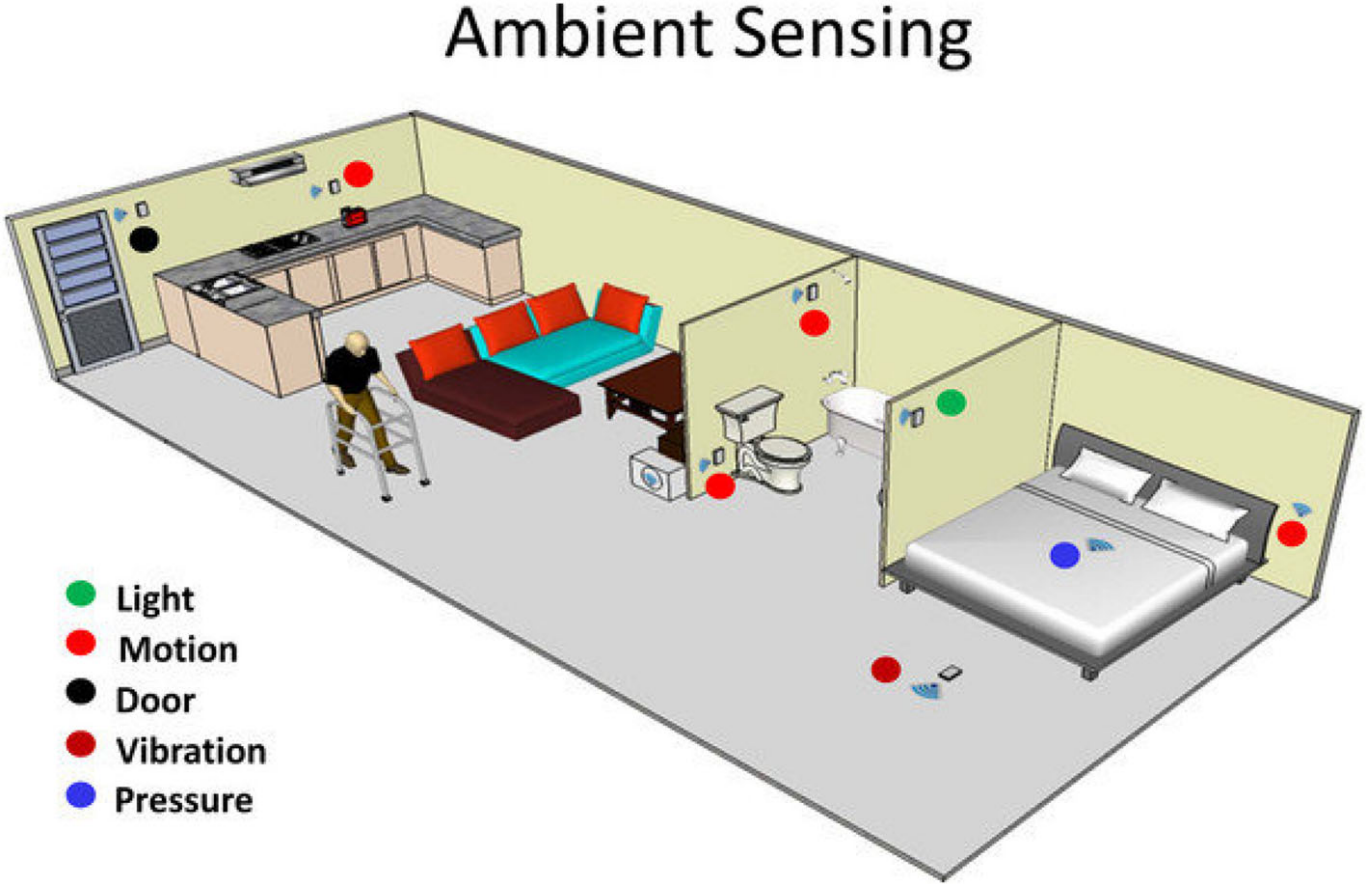
People are monitored by ambient sensors in an unobtrusive manner [94]

#### Implantable devices

Implantable electrical devices can be used for various diagnostic and therapeutic purposes in various medical fields. Implantable devices can be inserted into the bodies of patients. Bio-materials, a class of specialty polymers, are used to manufacture implantable medical devices to meet industry demand for metal replacements that give effective performance and fulfill the standards for extended or lifelong associated with biological fluids and tissue in the human body. Implantable devices are used for orthopedics, cardiology, spine, sports medicine, extremities, trauma, and more, as shown in Fig. 13. The following are examples of current devices:

- **Swallowable camera capsule:** It is difficult to detect a range of problems in the gastrointestinal system, especially in the small intestine. In the field of diagnosis, medical technology has made significant advances. The capsule endoscopy alters the diagnosis of the gastrointestinal tract dramatically. A camera, light bulb, battery, and radio transmitter are commonly included in the capsule used in capsule endoscopy. Peristalsis causes the capsule to move around in the body. The method is straightforward and does not necessitate any prior knowledge. When it comes to diagnosing lesions, Crohn’s disease, ulcerative colitis, and ulcers, capsule endoscopy has proven to be the best and most sensitive method for diagnosing lymphoma, carcinoid tumors, etc. [92]. The swallowable camera capsule provides at least two photos per second. The camera capsule is taken orally and expelled after 36–72 h. The battery in the swallowable camera capsule is charged and ready to use after excretion. Capsule endoscopy is a painless procedure that provides more convenient data than endoscopy. It visualizes the patient’s gastrointestinal tract from within.
- **Embedded cardiac implantable electrical devices (ECIEDs):** these are highly complex modern gadgets that offer patients a variety of benefits, including improved survival and quality of life. It collects data and delivers it through a radio connection to a nearby ubiquitous network. As listed below, three types of embedded cardiac implanted electrical devices (ECIEDs) are widely used.
  - **Implantable cardioverter defibrillator (ICD):** This battery-powered gadget is implanted beneath the skin and monitors the heart rate. An ICD device contains wires implanted into cardiac tissue that can provide electrical stimulation, monitor heart rhythm, and sometimes “rate” the heart’s beats as necessary.
  - **Pacemaker:** It is a little battery-operated gadget that improves with the regular beating of the heart. A transitory pacemaker may be required during hospitalization following a heart attack due to a slow heart rhythm; however, a pacemaker may not be required long term unless damage to the heart’s conduction system from the heart attack affects the ability to maintain a normal heart rhythm and rate.
  - **Cardiac re-synchronization therapy (CRT:) ** This system comprises two parts: the pulse generator, or device, and thin, insulated wires known as leads. A CRT device sends minute quantities of electrical energy to the heart through these leads. This helps to reestablish regular heartbeat timing, causing both ventricles to pump together more efficiently, such as a fist closing normally again.
- **Left ventricular assist device (LVAD): **This is a surgically implanted, battery-powered mechanical pump-type device. It aids in the maintenance of a heart’s pumping ability when it is unable to perform correctly on its own.
- **Implantable Nerve Electrodes (INEs): ** These are essential treatments for neurological diseases, such as epilepsy, Alzheimer’s disease, Parkinson’s disease, and others, particularly in the absence of specific medications [93]. INEs can investigate and regulate the nervous system by recording electrical nerve impulses or stimulating nerve tissue.

#### Ambient devices

Ambient sensors can unobtrusively monitor people in their homes. These devices monitor activity patterns, sleep quality, toilet visits, and other factors in the patient’s environment, and warn carers when aberrant patterns are detected. Such sensors are intended to make rooms more secure for individuals with chronic illnesses. One application is ambient assisted living (AAL), which leads to intelligent health-assistance systems in a person’s home. As shown in Fig. 14, AAL innovations are integrated (distributed across the whole surroundings or embedded into appliances or furniture), customized (made to fit the customers’ requirements), adaptive (adaptable to the individual and the patient’s environment), and preemptive (predicting users’ preferences as much as possible without conscientious mediation) [94]. The following are examples of ambient sensors:

**Fig. 15:**
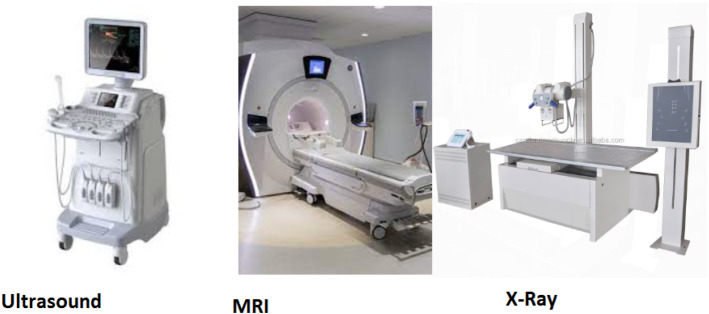
Stationary medical devices

- **Motion sensors:** A motion sensor is an electrical device that detects ambient motion using a sensor. A sensor is frequently used as part of a system that automates an activity or notifies a user of motion in a specific area. The two most popular motion sensor technologies are active ultrasonic and passive infrared (PIR) sensors, known for their accuracy and reliability.
- **Room temperature sensors:** Temperature sensors monitor the degree of heat energy or even coldness produced by an object, permitting one to “sense” or detect any physical change in that temperature, generating an analog or digital output. Temperature sensors come in various shapes and sizes, each with its features based on the application. A smart thermostat sensor, for example, is a wireless remote sensor that can monitor the temperature in multiple rooms and send the data to the central thermostat. A smart thermostat sensor offers homeowners more control over the heating and cooling of under-served areas of their homes.
- **Pressure sensors:** Pressure sensors are equipment or devices that convert the intensity of the physical pressure imposed on the sensor into an output signal that can be used to calculate the pressure’s quantitative value. Many distinct pressure sensor devices perform the same functions but use various underlying techniques to convert pressure to an output signal. These sensors measure the pressures of solids, liquids, and gases and water levels, fluid movement, speed, and height. Examples are compressors, pumps, mechanical ventilators, blood-pressure transducers, and other sensors.
- **Vibration sensors:** A vibration detector is a sensor that detects the frequency and magnitude of vibration in a system, machine, or system component. These metrics can identify equipment imbalances or other flaws and predict future failures. Vibration sensors are based on various operating principles, the most popular accelerometer-based. Vibration-based sensing systems for human and infrastructure safety and health monitoring are now available. These technologies use structural and body vibration as a data source and can be used in wearable and non-wearable devices. Moreover, the vibration detection technique uses low-cost, low-power sensors, making them suitable for outdoor and indoor monitoring. For example, it is used to monitor the movement of patients in their beds.
- **Daylight sensors:** These sensors detect natural light and alter the lighting zones in the space automatically.
- **Door sensors:** To prevent infection, monitor the status of the door (open or closed).

**Fig. 16:**
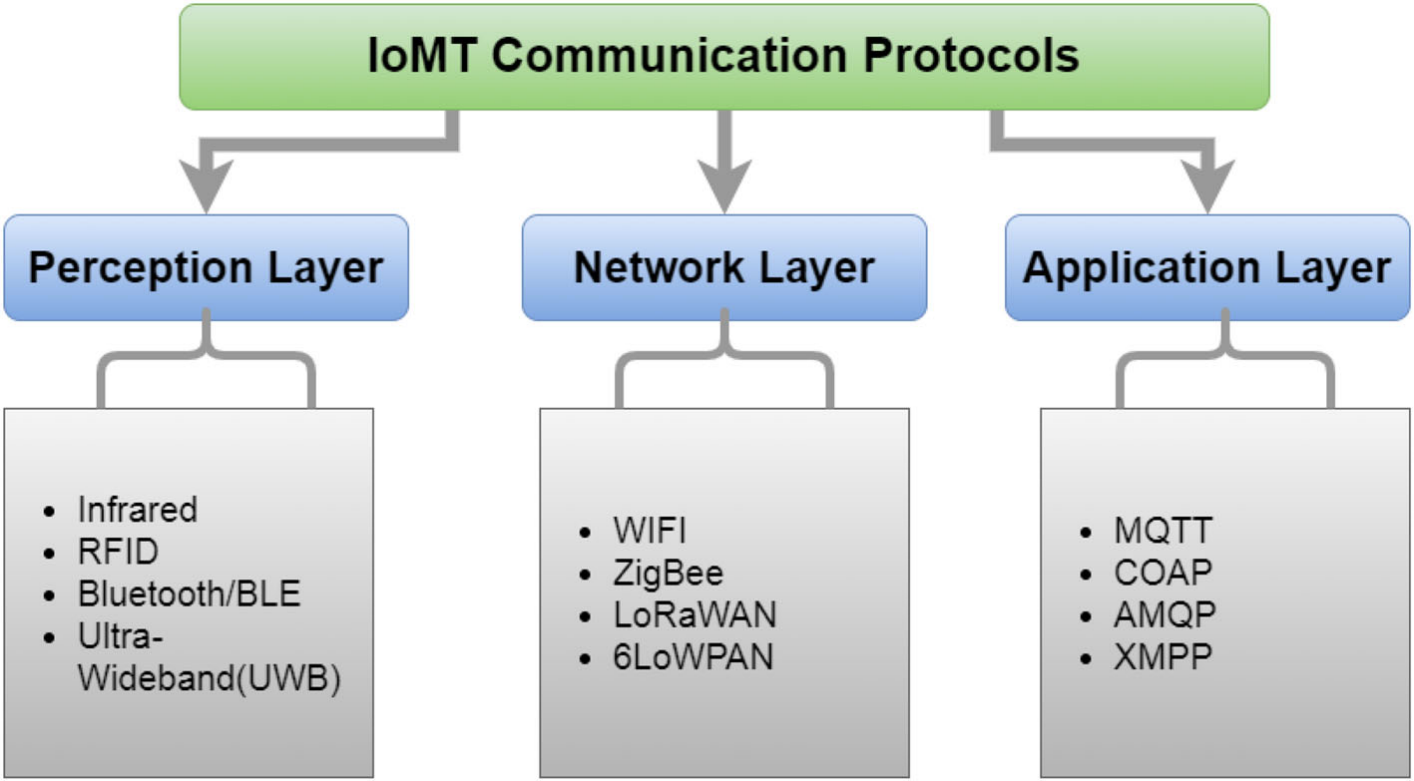
Taxonomy of communication protocols

#### Stationary devices

This group includes stationary equipment that the patient does not generally carry, as shown in Fig. 15.

- **Imaging equipment:** This is a process and technique of imaging the inside of a body for clinical examination and surgical intervention, as well as visualization of the function of particular anatomical structures (physiology). Medical imaging aims to expose hidden interior structures beneath the skin and bones and detect and cure disease. Medical imaging also creates a normal anatomy and physiology database, allowing anomalies to be detected. Although medical imaging of damaged organs and tissues is possible, such operations are normally classified as pathology rather than medical imaging. The following are imaging equipment examples that provide visualizations of the inside of a body for clinical research and medical treatment.
  - **Computed tomography (CT):** This scan is an effective tool for detecting diseases and injuries [95]. It creates a 3D image of soft tissues and bones using a sequence of X-rays and a computer. A healthcare professional can diagnose diseases using CT scans, which are painless and noninvasive. A CT scan can be done in a hospital or imaging center.
  - **Magnetic resonance imaging (MRI):** A high magnetic field, radio waves, and a computer are used to produce comprehensive images of the inside of your body in magnetic resonance imaging (MRI). MRI is a non-invasive imaging technique that creates comprehensive three-dimensional anatomical images. It works by stimulating and detecting changes in the direction of the rotating axis of protons in the water that makes up living tissues. It is frequently employed in detecting, diagnosing, and monitoring diseases. It can diagnose or track treatment progress for several disorders affecting the chest, abdomen, and pelvis.
  - **X-ray:** It is a painless, rapid test that generates images of the inside components of the body, especially bones. X-rays use small amounts of radiation to create images of the body. X-ray beams penetrate through the body, and depending on the density of the material, they pass through and are absorbed in varying amounts. On X-rays, dense materials, such as bone and metal appear white. The lungs’ air appears to be black. Fat and muscle appear as grayscale images.
  - **Ultrasound:** This is an imaging technique that employs sound waves to create a picture of organs, tissues, and other structures inside the body. An ultrasound can also reveal moving components of the body, such as a beating heart or blood flowing through blood arteries. Ultrasounds, unlike X-rays, do not employ radiation.

### Overview of IoMT communication protocols

Data transmission for the IoT network has been enabled using a variety of protocols. The purpose of the protocol in use is to determine how an object behaves during data transfer [96]. It comprises a syntactic and semantic set of rules that determine the computer network’s operational activity block during data transfer [28]. Unfortunately, standard Internet protocols (IPs) are insufficient for assuring good data sharing. It is challenging to design an IoT network, because it includes several sensors that cannot be integrated into the generic address format, restricting the ability to gather a properly functioning node.

Furthermore, these nodes rely on a consistent energy supply, storage characteristics, and channel throughput capacity, necessitating effective resource management. In the case of Wireless Sensor Networks (WSNs), a data sink must be added to the network [97]. The procedure entails first putting the acquired data in the sink, then moving on to the other nodes and repeating the procedure [98]. Because the placement of sensors and sinks can increase the capacity of an IoT network, the appropriate choice of a data transmission technique has a significant impact. It guarantees security and privacy by preventing numerous sensors from sending and receiving the same data. It conserves energy [99, 100]. Medical-specific IoT communication protocols are divided into three groups based on the five-layered IoMT architecture: perception, network, and application layer protocol, as illustrated in Fig. 16. The characteristics of the perception and network layer protocols used in IoMT are listed in Table 5. It also includes IoMT-specific examples that show how each protocol might be used in the health sector.

**Table 5:** Summary of IoMT perception and network layer protocols

IoMT protocols	Layer	Standard	Data-rate	Transmission range	Frequency	Cost	Power consumption	Topology
Infrared	Perception-network	Not standardized	14.4 Kbps	1 m	850–900 nm	LoW	Low	LAN
RFID	Perception	ISO, IEC	106–424 Kbps	20 cm	13.56 MHz	Low	Very low	Ring
Bluetooth	Perception-network	802.15.1	1–24 Mbps	8–10 m	2.4 GHz	Low	Medium	Star
Ultra-Wideband	Perception	802.15.4	53–480 Mbps	10 m	3.1–10.6 GHz	Low	Low	Radio technology
Wi-Fi	Network	802.11a, b, g, n	2–54 Mbps	20–100 m	2.4 GHz	Low	High	Star
ZegBee	Perception-network	802.15.4	20–250 Kbps	10–20 m	868/915 MHz, 2.4 GHz	Low	Low	Star, Tree, mash
LoRaWAN	Network	802.15.4g	0.3–50 Kbps	3–4 km	133/868/915 MHz	Low	Very low	Star
6LoWPAN	Network	802.15.4	250 kbit/s	10–100 m	2.4 GHz	Low	Low	Star

#### Perception layer protocols

The majority of perception layer protocols use the IEEE 802.15.4 standard. IEEE 802.15.4 is a low-complexity, low-cost, and low-power networking standard. It primarily offers low-bandwidth IoT devices and wireless connectivity with a significantly lower data rate.IEEE 802.15.4 defines the physical (PHY) and media access control (MAC) layers for a variety of devices (fixed, portable, and mobile) that require little or no power. The Direct Sequence Spread Spectrum (DSSS) approach generates a wide-band physical layer. Physical layer devices are controlled in three frequency bands: (a) the 915 MHz band, which is licensed in the United States; (b) the 868 MHz band, which is licensed in Europe; and (c) the 2.4 GHz ISM band, which is an unauthorized band widely available. Through these three bands, there are 27 broadcast channels. The IEEE 802.15.4 physical layer is in charge of various low-level functions, including data transmission and reception, channel energy sensing, signal strength, clear channel analysis, etc. The IEEE 802.15.4 data link (MAC) layer is in charge of establishing the Personal Area Network (PAN), Carrier Sense Multiple Access with Collision Avoidance (CSMA-CA) for channel allocation, Guaranteed Time Slot (GTS) data transmission, control repeater transmissions, and reliable link establishment between peer entities, as well as ensuring synchronization with the repeaters. Numerous IoMT protocols, including ZigBee, Bluetooth, and LowPan, are adequate with IEEE 802.15.4. Healthcare systems have employed the perception layer protocol detailed below to obtain medical data from sensors.

- **Bluetooth:** This is a wireless communication system that employs ultra-high frequency (UHF) radio waves to communicate across short distances. This technology provides wireless communication between multiple parts of medical equipment. Bluetooth operates on the 2.4 GHz radio band. Bluetooth can communicate up to 100 m apart. Bluetooth secures data using encryption and authentication. The primary advantages of Bluetooth are low cost and power efficiency. It also ensures less interference between connected devices during data transfer. However, when it comes to long-distance communication in a healthcare application, this technology falls short [101].
- **RFID:** It comprises a system of assigning a unique identifier to each object uniquely in record data. RFID comprises readers, hosts, and tags, each absorbing and emitting radio waves, offering the term responder. In various sizes and forms, active and passive RFID tags are marketed. Compared to active tags, passive tags are more valuable, because they are less expensive. Tags contain a unique ID number and climate data, such as moisture content, temperature, and humidity, among other things. To recognize an object, these tags are integrated and connected to it. The mentioned frequency ranges are generally used in RF communications: low frequency—125 kHz, high frequency—13.56 MHz, ultrahigh frequency—433 MHz, and 860–960 MHz and microwave—2.45 GHz, 5.8 GHz. Intelligent RFID tags deployed inside or near a patient’s body are essential supporting technologies for establishing smart medical systems that are entirely transparent to the patient. Furthermore, passive RFID tags can be used to monitor the environment of patients, and healthcare organizations can deploy RFID cards to secure physical entrances.
- **Infrared:** This communication is one of the most basic wireless communication technologies, and it is a cost-effective way of conveying a few bits of data remotely. It is safe to assume that anybody reading this has used a variety of remote controls at home or work, and the majority of those simple gadgets interact with the receiver via infrared pulses. The NEC protocol uses pulse distance encoding to distinguish between logic states in transmitted messages. A synchronization sequence, an eight-bit address, and a command comprise the message itself. The RC5 protocol uses Manchester encoding to distinguish between logic states. The message consists of two start bits, a toggle bit, five address bits, and six data bits. Both systems support repeat sequences that prompt the user to execute the previous command again. Infrared is employed in thermometers and cameras in the medical domain. Thermal imaging technology based on infrared light is also used to analyze and measure the temperature of a body.
- **Ultra-wideband (UWB): **This is also known as an ultra-wideband, or ultra band, and is a wireless communication protocol for short ranges. It employs radio waves to allow devices to communicate with one another. It also employs a higher frequency. Ultra-wideband has a frequency range of 3.1–10.6 GHz. One disadvantage is its small range, but this is not a big deal when one has two devices in the same room. A UWB transmitter sends billions of radio pulses across a broad spectrum of frequencies, subsequently translated into data by a UWB receiver. In the same way that bats use auditory signals to perceive their environment, UWB pulses may be used to measure distances between two transmitters. The more accurate the distance measurement, the shorter the impulse period. Since it sends up to 1 billion pulses per second, UWB achieves real-time precision (about 1 per nanosecond). Ultra-Wideband is a radio protocol that is rapid, reliable, and low-power, and it is used to detect locations with greater accuracy than any other wireless technology. For example, the ECG method needs short-distance communication technology, and UWB, such as other protocols, has been used for this function.

#### Network layer protocols

The network layer comprises infrastructure, such as gateways, access points, and routers and is responsible for internet protocol (IP) addressing and other network functions. The IEEE 802.15 standard serves as the foundation for most of the protocols at this layer. At this layer, the two most popular protocols for IoMT are WiFi and ZigBee. LoRaWAN and 6LoWPAN are two other protocols implemented in medical systems for communicating with wireless sensor networks. Standard cellular wireless communications technologies, such as GPRS or 3/4/5 G, can be used for remote data transmission and communication.

- ** WiFi: ** IEEE 802.11 is a group of wireless local area network communication standards, including 802.11a, 802.11b, 802.11g, 802.11n, and 802.11ac. These standards all work at different bandwidths of 5 GHz, 2.2 GHz, 2.4/5 GHz, 60 GHz, and 5 GHz. These standards’ data transfer rates range from 1 Mb/s to 7 Gb/s. It has a communication range of 20 to 100 meters. Many researchers have suggested using WiFi to communicate between remote monitoring and control devices in an IoMT system. For example, WiFi networks have been deployed on medical equipment, including defibrillators, infusion pumps, anesthetic machines, and lung ventilators. It is proven that WiFi can be used efficiently and securely for device communication.
- **LoRaWAN:** This is an abbreviation for long-range chirp spread spectrum (CSS)-based spread spectrum modulation. Lora is a low-power, long-range wireless network that has emerged as the unofficial wireless platform for the IoT. LoRaWAN devices and networks enable smart IoT applications that address some of the world’s most important concerns, such as infrastructure efficiency, energy management, pollution control, natural resource conservation, and disaster avoidance. LoRa devices are used in smart cities, houses and buildings, communities, metering, supply chain, logistics, agriculture, and other applications [29, 102].
- **Zigbee:** This is a popular protocol for connecting medical devices and transferring data. Zigbee has a comparable frequency band to Bluetooth (2.4 GHz). However, compared to Bluetooth devices, it has a greater communication range. A mesh network topology is implemented in this technology. End devices, routers, and a processing center are the components of the system. The processing center handles the data analysis and aggregation. The mesh network keeps the rest of the devices connected even if one or two of them fail. Power dissipation, rapid transmission rates, and massive network throughput are just a few of the advantages of Zigbee [103].
- **6LoWPAN:** Its abbreviation is IPv6 over Low-Power Wireless Personal Area Networks. It is a low-power wireless mesh network with individual IPv6 addresses for each node. This enables the node to connect to the Internet directly using open standards. The idea behind 6LoWPAN was that the Internet Protocol should be applied to even the tiniest devices and that low-power devices with limited compute capability should be permitted to participate in the IoT. The 6LoWPAN has specified encapsulate and header compressing techniques that help standard IPv6 packets be transmitted across IEEE 802.15.4-based networks. Furthermore, the 6LoWPAN IETF team has created a protocol called 6LoWPAN over Bluetooth Low Energy (RFC 7668).In the medical field, IoMT sensors and remote devices can be communicated to IP networks using 6LoWPAN, enabling sensor connectivity. In addition, it enables sensor interaction with middleware systems or network routers.

#### Application layer protocols

Table 6 presents the application layer protocols used in IoMT and healthcare applications, as well as essential implementation details. These communication is required to keep the devices connected. In addition, numerous communications protocols, as mentioned in Table 7 are being developed to facilitate and simplify the usage of IoT. The most commonly used messaging protocols are the Message Queue Telemetry Transport (MQTT) and the Constrained Application Protocol (CoAP). However, other well-known protocols, such as the Extensible Messaging and Presence Protocol (XMPP) and Advanced Message Queuing Protocol (AMQP), contribute to overall network efficiency.

**Table 6:** Summary of IoMT application layer protocols

IoMT application protocols	Standards	Architecture	Encoding format	Header/message
MQTT	IETF, Eclipse foundations	Client–server, Broker	Binary	4 Byte/small
COAP	OASIS, Eclipse foundations	Client–broker	Binary	2 Byte/small
AMQP	OASIS AMQP TC	Publishers-subscribers, Broker	Primitive, or a described format code	Undefined, large
XMPP	IETF, open standard	Client–server	Binary	1023 bytes

- **CoAP:** This protocol is implemented in the IoT communication loads that are sensitive to traffic congestion-induced performance degradation. It is a web transport protocol with a low bit rate designed for devices with limited processing power and memory [104]. HTTP is a hypertext transfer protocol that allows the RST architecture to be extended to Low-Power Wireless Personal Area Networks (LoWPANs) [105]. Moreover, The Medium Access Control (MAC) mechanism is also provided by the Low-Power Wide-Area Network (LoRaWAN) protocol, facilitating communication among different devices and networks. This protocol is designed with a star topology and offers numerous benefits in IoT applications, including low cost, power consumption, security, and easy implementation. The User Datagram Protocol (UDP) is the primary transport layer protocol. It is based on the RST architecture and has a 4-byte header-only format [106]. The re-transmission timeout method [107] also provides dependability. Because it is built on top of UDP, CoAP assumes end-to-end trustworthiness and main congestion control. At the application layer, this protocol is responsible for arranging the data formatting handshaking communication [108]. The acknowledgment message, the confirmable message, the non-confirmable message, and the reset message are all used by CoAP to send data. The request/response paradigm is the foundation of CoAP [109].
- **MQTT:** This is an asynchronous protocol that is used for light machine-to-machine communications and leverages the publish/subscribe principle. This protocol’s primary function is to connect embedded network systems to middleware and applications. MQTT benefits include the guarantee route in small places and low memory, low cost, and low-power equipment for susceptible and low-bandwidth networks. Because this protocol is lightweight, it is better suited for WSN, M2M, and IoT applications [110]. It allows high-latency or limited networks for data transmission from devices to the server in the style of telemetry [111].
- **XMPP:** This protocol is mainly used for sending and receiving messages. In contrast to the CoAP Request/response approach, it employs the publish/subscribe mechanism, which is better suited for the IoT. Furthermore, despite the availability of newer protocols, such as MQTT [112], it is an early internet protocol that has been supported. It is based on the Internet Engineering Task Force (IETF) standards for cross messaging, telepresence, and video and audio calling [113]. The key advantages of XMPP are that it is a reliable protocol and that it enables the development of additional applications [114].
- **AMQP:** was designed with the business industry in mind. Its capabilities include message orientation, switching, queuing, security, privacy, and reliability [115]. The AMQP protocol, such as XMPP, is built on a publish-subscribe model. This protocol ensures that messages are sent successfully and that delivery primitives, such as at-most-once, at-least-once, and precisely once are employed. The main advantage of using AMQP is the store-and-forward feature, which assures dependability and trustworthiness. However, it may create network interruptions [116]. It requires a reliable transport protocol that specifies how it uses the Transmission Control Protocol (TCP) to deliver and receive messages [117].

**Table 7:** Overview of communication protocol in IoMT applications

References	Protocol	Applications
[104]	COAP	Traffic congestion increases IoT communication load
[105]		RST should be expanded to include LoWPANsg
[106]		Reliability is achieved by the use of re-transmission mechanism
[107]		Application layer
[108]		Formatting handshaking connection
[118]	MQTT	WSN,M2M, and IoT
[110]		Lightweight M2M communication
[111]		Telemetry-style data transmission
[112]	XMPP	Security
[113]		Exchange of messages
[114]		Reliable and trustworthy network
[115]		Multi-party chatting, telepresence,and voice & video calling
[116]		Financial industry
[117]		TCP for exchanging messages
[119]	LoRa	Collecting human body data, “MySignals” developed a healthcare management solution based on the LoRa wireless network

## IoMT security and privacy

A recent study [29] claims that more than 90% of all IoT device traffic is unencrypted, which means that 57% of IoT devices are susceptible to attacks that reveal sensitive data. Cyber-attacks are not only disruptive to the system; they may even endanger people’s lives. Any Cyber-attack has the potential to be devastating, putting patients’ lives at stake [120]. The rapid expansion and adoption of IoMT, particularly during pandemics, may raise ethical security issues, making it more challenging to secure the privacy of crucial and sensitive medical data. Several attacks, threats, and hazards can have an impact on various levels of the IoMT architecture [120].

**Fig. 17:**
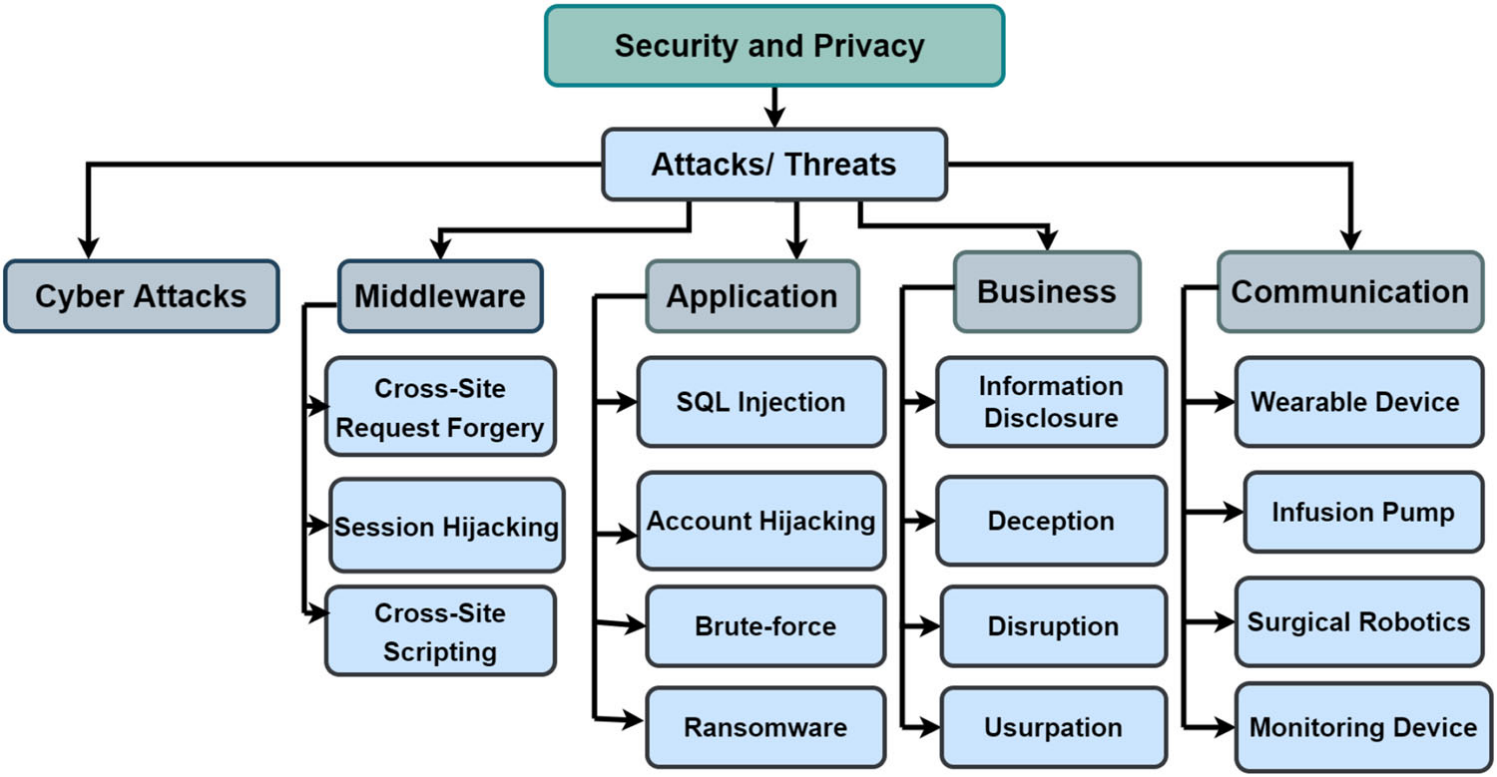
Taxonomy of IoMT security and privacy

As a result, the IoMT industry must follow rigorous security and privacy restrictions. It was also noted that the IoMT has security and privacy issues, as illustrated in Fig. 17, that must be addressed. For effective intrusion detection and prevention, methods such as cryptographic or non-cryptographic algorithms are recommended. Various malware attacks targeting IoMT systems have been discovered to compromise data security, integrity, authenticity, and data availability. Key management, intrusion detection, authentication, and access control have all been prioritized in the current security approach [121].

### Attacks on IoMT middleware

In today’s IoMT environment, cloud-based technologies are widely used [122]. This layer collects and filters data from perception layer equipment (sensors), discovers services, and controls device access [123]. A Hewlett–Packard (HP) investigation [121] claims that session hijacking and cross-site request forgery (CSRF) breaches have been discovered in numerous IoT projects, affecting 60% of all IoT devices connected to the cloud. The following are the attacks on this layer:

- **Cross-site request forgery:** This form of attack is becoming more frequent on RESTful API-enabled IoT devices. The CSRF approach deceives the end-user into responding to a susceptible application without awareness. If not set appropriately [124], the IoT layer’s web interface becomes vulnerable to CSRF attacks.
- **Session hijacking:** This type of attack is popular in RESTful-based IoT devices. Sessions could be hijacked due to numerous IoT devices maintaining session connectivity at the web application interface, allowing an attacker to access session data.
- **Cross-site scripting:** XSS also takes advantage of RESTful IoT apps by injecting side scripts onto web pages to evade access restrictions. Such attacks are enabled via the websites of cloud-connected IoT applications [125].

### Application layer attacks

Since the emergence of clouds, application providers have turned more toward hosting programs in the cloud due to enhanced scale and adaptability. This layer is the user-friendly interface for IoMT gadgets, connecting people through a middleware layer. The following are examples of possible attacks on this layer:

- **SQL injection:** A SQL injection vulnerability in a cardiac management system has been discovered. This attack occurs when an attacker injects an incorrect SQL query into the application’s backend database. A powerful SQL injection attack might breach or change confidential patient records, posing a significant threat to IoT devices, particularly in the health industry [121].
- **Account hijacking:** Several IoT devices use insecure encryption or communicate in text format over the Internet. Intercepting the packet when an end-user has been authenticated allows an attacker to undertake account hijacking. As documented in several situations [126], the primary source of the creation of this attack is ancient operating systems with unpatched vulnerabilities.
- **Brute-force:** this comprises exploring every possible option to guess inputs, such as passwords. IoMT apps are vulnerable to brute-force attacks, because there is inadequate security to prevent such attacks on IoT devices. This is due to the simulated computing capacity of the sensors. Pacemakers are particularly sensitive to this type of attack [127].
- **Ransomware:** encrypts crucial data and demands much cash to restore it. This threat could start on a single machine and propagate throughout the network. Attackers can encrypt confidential information, such as patient information, and keep the decryption code in exchange for cash [128].

### Cyber-attacks

**Fig. 18:**
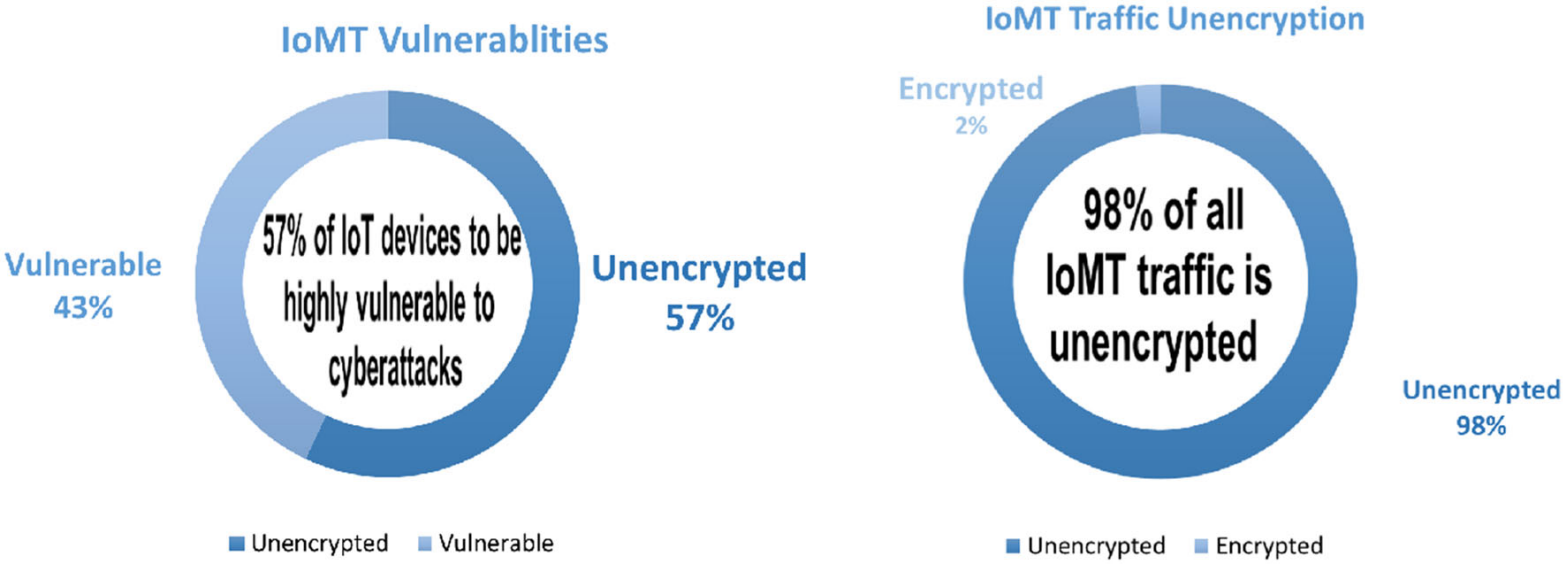
Cyberattacks in IoMT

With the emergence of connected autos, smart cities, and next-generation health gadgets, hackers may target devices that bridge the digital and physical worlds rather than steal private data in the virtual world [129–131]. Devices connected to the IoMT are particularly vulnerable entry points for hackers, and health information could be compromised. According to a recent study of IoT medical imaging devices, 83% are running unsupported operating systems. Healthcare institutions that utilize these devices may become more exposed to cyberattacks that reveal sensitive medical data  [29, 132, 133]. As shown in Fig. 18, 98% of all IoT device communication is unencrypted, leaving 57% of IoT devices exposed to cyberattacks and exposing personal and private data on the network [29]. It should be anticipated that cyberattacks will increase in frequency and severity with the development of connected devices.

### Business layer threats

This layer manages the healthcare provider’s business logic and enables the business process life-cycle, including monitoring, management, and improvement. It is also in charge of gleaning insights from IoMT data. Attacks on this layer have been explored previously, but they have a higher impact, since they contain sensitive medical information. Information disclosure, deceit, disruption, and usurpation are possible attacks.

- ** Information disclosure:** The security of the IoMT system is threatened by unauthorized access to sensitive data, such as medical records. To gain unauthorized access to sensitive information, an attacker could employ previously outlined techniques, such as session hijacking and CSRF. In 2021, this type of breach accounted for almost 38% of all healthcare breaches [134].
- **Deception:** Data integrity is harmed by corrupted data, which can have negative impacts. Information deception may be caused via attacks, such as sinkholes and man-in-the-middle. Approximately 58% of institutions lack a mechanism for correcting incorrect information [135].
- **Disruption:** The availability of the system is harmed when correct operations or access to medical information are interrupted, which might have life-threatening implications. A DoS attack is an example of a cyber-attack that tries to disrupt information [136].
- **Usurpation:** The integrity of medical equipment is affected by unauthorized access to specific areas of the system by attacks, such as sinkhole, replay, and code injection [126].

### Threats to IoMT communication layer

Every malicious action attempts to access a medical device using communication layer protocols to destroy equipment, steal medical data, or render it unusable.

- **Wearable device attacks:** Wearable devices typically interface with other devices, such as smartphones, via a communication protocol (such as Bluetooth Low Energy) or an accumulator, for example, to gather medical information from a variety of sensors. In this case, a smartwatch serves as a pulse oximeter, and it is connected to a smartphone through Bluetooth Low Energy (BLE). The attacker may be close enough to connect with the wearable device via force. The information captured by a person’s phone can be retrieved by the attacker’s phone [137]. This attack relies on the fact that the wearable device and the smartphone need not verify each other at each connection point. As a result, the wearable device cannot distinguish between the smartphone of the genuine user and that of the attacker. To avoid such threats, keep your authentication credentials up to date, avoid auto-pairing, and only pair with authorized devices [138].
- **Infusion Pump Attacks:** The infusion pump is a type of therapeutic equipment. It may be controlled remotely or with direct access by the patient, so a doctor or nurse can use it in either mode. Below are several real-life examples of proven susceptible infusion pumps used in healthcare settings and potential concerns. The US Federal Drug Administration (FDA) prohibited the sale of a certain infusion pump in 2015 to prevent accidents. Researchers uncovered many flaws in a certain model of Hospira infusion pumps [139]. Because of these flaws, a hacker was able to gain access to the pumps and change the amounts of drugs that were supposed to be dispensed. The FDA advised all hospitals in California and around the country to stop using potentially dangerous medical equipment [139]. Because infusion pumps may be accessed physically, human error and physical manipulation are potential concerns. Strong identification and authentication are necessary to prevent an attacker from gaining access to the device.
- **Surgical robotics assaults:** Direct attacks on interconnected surgical robots or external attacks on ambient equipment such as gyroscope sensors that could impact a surgical process are real attack possibilities. Micrometer precision is required due to the nature of the technique. It is possible to attack the surgical robot directly or indirectly through the sensors. Perception layer attacks are the most common attack on gyroscope sensors. By sending out signals, an attacker can use replay attacks to mislead the actual gyroscope signals. As a result of this attack, the mapping of the human body may be harmed. It has the capability of changing coordinates or generating error messages [140]. To successfully undertake such an attack, the attacker must be in close proximity to the sensor. As a result, the doctor must work outside the operating room, free of tracking and identification technology. Attacks such as the ones listed below are possible in the event of a direct attack on the surgical robot.
  - **Changes to the robot’s intent:** The attacker can alter packets, while they are being transported. This activity may result in small device problems, such as odd robot motions or delays.
  - **Controlling the robot’s intent:** In this scenario, the intruder is unable to manipulate the medical equipment, but he or she may have an impact on the device’s feedback, such as pictures and coordinates.
  - **Hijacking:** The invader manages to seize control of the surgical robot.
- **Monitoring device attacks:** A suspicious user might try to hijack a camera or disable an alarm system in this situation. If anybody tries to interfere with a treatment device’s alarm system and the alert is turned off for a few minutes (or even seconds), a patient may receive a pharmaceutical overdose with catastrophic consequences. Attack patterns include denial-of-service attacks and SQL injections. Strong authentication systems and advanced intrusion detection systems (IDS) may be required to protect against such attacks [141]. The importance of early identification is well-acknowledged [142]. Indeed, it is mainly concerned with application layer protocols, such as MQTT and COAP.

On the other hand, many hackers have malevolent motives, increasing their numbers. Honeypots are decoy servers used by cybersecurity organizations to evaluate trends and patterns. Honeypots, set up worldwide, can be attacked by malware-infected smartwatches or linked toothbrushes. According to security experts, honeypots recorded a 446% rise in attack traffic in 2019, with hits jumping from 1.0 billion to 5.7 billion [143].

## IoMT research open concerns and future directions

The mechanisms of grouping complex systems that synchronize and work collaboratively and simultaneously to seem like a centralized platform to the end-user are known as “systems of systems.” Internal systems combine their resources and competencies to provide further services as a unified system. One example of a system of systems is smart healthcare. Since the recent pandemic, COVID-19, is considered, this emphasizes a tremendous area of research, as no such preemptive strategy had been discovered at the start [144]. The integration of IoMT could lead to an extensive range and complexity of cutting-edge technology-based healthcare facilities and medical procedures that were initially assumed unattainable before the nano-tech generation. Designing, developing, and operating such nano-sized devices effectively is a concern that requires great consideration to enable humans to live healthier lives in the long run. Therefore, developers must be familiar with smart healthcare systems’ significance and real-world implementation to respond to the demands and cope with smart healthcare. A comprehensive range of research gaps and constraints must be addressed for such a broad range of diverse research and development in smart healthcare systems. Smart healthcare architectures and frameworks are being developed by researchers to deploy, coordinate, and develop smart IoMT applications. Moreover, with the emergence of various technological advancements, such as cloud computing, the IoMT security and privacy community has yet to extensively explore a variety of promising future research fields. Some research directions for strengthening the healthcare systems are listed below.

1. **Advanced communication technologies ** Potential users will be highly dedicated to smart healthcare systems in terms of the benefits provided by emerging advanced communication technologies. However, technological advancements in recent years have led to rapid advancements in processing. It demands a quick and efficient network connection to maintain system synchronization, which demands the development of 6G networks. Future intelligent healthcare networks are expected to combine 6G and IoT technologies to increase device integration network efficiency and resolve security concerns. To provide well-satisfied smart healthcare, 6G technology advancements, such as SDN, massive MIMO, NFV, M2M communication, and mm-wave communication are critical to overcoming the aforementioned issues.
2. **Big data** The processing of a massive volume of medical data is one of the most significant concerns of smart healthcare. This data is generated by IoT sensors that are implanted in human bodies, so as a result, the patient’s health state is constantly changing. In addition, as the need for accurate and consistent physical examinations evolves, sensors and access points generate many types of medical data in various formats. Working with such diverse and considerable amounts of data may hinder access to this data at any time, even though uninterrupted accessibility is supplied. The repercussions for some essential patients and critical situations may be devastating. Remote monitoring healthcare equipment must alert the patient’s doctor or caretaker in critical situations such as low blood pressure, rapid changes in heart rate or even a fall of an elderly person [145]. Significant data capacity fluctuations will surely decrease in the foreseeable future, because such instances can be identified. According to the International Institute for Analytics, organizations will most likely collaborate with internal and recruitment training for aspiring scientists to get their challenges resolved. Corporations will soon obtain computations rather than system procedures and update their data. More corporations will seek to generate profits from their data. Existing services and suppliers, such as Kaggle, Algorithmia, and DataXu, will likely grow to vast proportions; as a result, algorithm economies will most likely emerge.
3. **Artificial intelligence**: Machine learning and deep learning [146] are the most prominent research subjects in practically every domain, such as network security. Recently, several machine learning-based networks, such as intrusion detection methods, have been proposed, and they may also be employed in IoMT healthcare systems. In [147, 148], deep learning networks were used to investigate the deployment of PHI in different tiers of IoMT systems for intermediate attack detection. Because deep learning techniques are increasingly being employed in medical servers for illness detection, they should also be evaluated for system security and privacy [149].
4. **Block chain** The blockchain was initially designed to secure sensitive transactional data decentralized, with interconnected “blocks” in the blockchain. It might be widely used in medical data dispersed across medical servers, providing significant security and privacy protection to IoMT medical systems. To build blocks, blockchain demands a substantial amount of computational resources on the devices, which is not viable on resource-constrained IoMT devices. In addition, blockchain might be used to secure digital medical records stored on cloud servers. For example, MedRec [150] is a pioneering study on using blockchains for medical data access and authorization control.
5. **Security assessment** There is no criterion for assessing the proposed IoMT security strength, because various research organizations independently conduct security research. Researchers perform adversarial analysis to determine the level of security in their research. However, these adversarial assessments cannot be comparable, since they are not predicated on the same suppositions and principles. As a result, the IoMT privacy and security community must develop a methodology for quantifying the standard of security in security research. IoMT-SAF [151] is an example of research. It is a web-based IoMT security assessment system that may provide ideas based on user input. On the other hand, this paper does not analyze the security of current studies or provide crypto-analysis for cryptographic methods. More research is needed to measure the security strength of IoMT healthcare systems.
6. **Medical robotic systems** The implications of the COVID-19 epidemic have highlighted the importance of patient protection and the need for medical robots to provide smart healthcare services. As a result, next-generation robotics and systems engineering will need fundamental, adaptive, simplified, and substantially less expensive technology. The digital replacement of tiny electrical controllers, sensors, and networking computers can make technologies more ubiquitous. Traditional electrical motors, such as drive trains and terminal motors, are not well suited to logical technology, despite the reality that they can be complicated, bulky, and expensive. Because these sorts of robotics were not identified, they were associated with significant challenges and limitations. Therefore, in the coming years, the most recent trend, the Internet of Everything, will undoubtedly be combined with the technology and equipment that we use in our daily lives to help us accomplish our jobs efficiently.
7. **Eco-sustainable technologies** In medicine, the study aimed to examine the mineral composition of drugs and investigate their adverse effects on modern smart healthcare systems [145]. Moreover, nanotechnology, particularly IoMT devices, can play a critical role in smart healthcare to make it more robust and dynamic. This field has an extensive research gap to close to establishing intelligent, smart environmental protection and enhancement system that relies on IoMT sensors.

## Conclusions

This survey paper provides an in-depth, comprehensive, and systematic review of IoMT in technical development, worldwide IoMT adoption, supportable market devices, applications, communication protocols, research gaps, and the most fundamental IoMT security and privacy challenges. First, an overview of the IoT and global IoMT underlying technologies, market trends, methodologies, and IoMT applications in many fields has been presented. Then, minor and significant similarities in related work reviews that have been done over the last few years and the contribution of each endeavor have been addressed. Furthermore, the relevance of security in the context of IoMT and how it differs from other systems due to the heterogeneity of its various applications has been emphasized. Furthermore, the most appropriate application fields and several valuable research benefits have been identified. This research paper gives complete knowledge to researchers and professionals in the field, assisting them in understanding the immense potential of IoT in the medical domain and identifying major IoMT applications and challenges.

During the COVID-19 pandemic, most IoMT systems were utilized primarily for tracking and identifying infected people as a form of digital monitoring, raising specific concerns about IoMT devices, applications, and communication protocols. However, many citizens around the world accept this as a necessary measure. Other IoMT applications have included infection detection, surveillance, and testing to reduce the infection risk or speed up diagnostic tests. IoMT systems are subject to the same security constraints as IoT systems, but the concern is higher, because IoMT devices affect people’s lives. As a result, this paper has thoroughly covered current achievements in IoMT security, including experiments using the latest technologies, such as blockchain to reduce security risks to individuals and systems. According to a discussion on the future direction of IoMT in the final section of this paper, hybrid technologies will be devised in the coming years, combining knowledge from the fields of big data analytics, data mining, artificial intelligence, and IoMT advances have made automation, modeling, and forecasting systems more effective and precise.

## Data Availability

Data sources are highlighted in the paper.
