# Correction to: A survey on COVID-19 impact in the healthcare domain: worldwide market implementation, applications, security and privacy issues, challenges and future prospects

**DOI:** 10.1007/s40747-022-00888-2

**Published:** 2022-10-17

**Authors:** Tanzeela Shakeel, Shaista Habib, Wadii Boulila, Anis Koubaa, Abdul Rehman Javed, Muhammad Rizwan, Thippa Reddy Gadekallu, Mahmood Sufiyan

**Affiliations:** 1 School of System and Technology, University of Management and Technology, Lahore, Pakistan; 2 Robotics and Internet of Things Lab, Prince Sultan University, Riyadh, 12435 Saudi Arabia; 3 Department of Cyber Security, PAF Complex, E-9, Air University, Islamabad, Pakistan; 4 Department of Computer Science, Kinnaird College for Women, Lahore, Pakistan; 5 School of Information Technology and Engineering, Vellore Institute of Technology, Vellore, India

## Correction to: Complex & Intelligent Systems 10.1007/s40747-022-00767-w

In the original article, there is a citation for the retracted article https://link.springer.com/article/10.1007/s10916-018-1045-z that will be removed.

The original article has been corrected.

